# Effects of Differently Processed Tea on the Gut Microbiota

**DOI:** 10.3390/molecules29174020

**Published:** 2024-08-25

**Authors:** Zimo Zhao, Ruofan Chen, Ken Ng

**Affiliations:** School of Agriculture, Food and Ecosystem Sciences, Faculty of Science, The University of Melbourne, Parkville, VIC 3010, Australia; zimozhao@student.unimelb.edu.au (Z.Z.); ruofchen@student.unimelb.edu.au (R.C.)

**Keywords:** tea, gut microbiome, tea polyphenols, animal feeding trails, human fecal fermentation, human feeding trials

## Abstract

Tea is a highly popular beverage, primarily due to its unique flavor and aroma as well as its perceived health benefits. The impact of tea on the gut microbiome could be an important means by which tea exerts its health benefits since the link between the gut microbiome and health is strong. This review provided a discussion of the bioactive compounds in tea and the human gut microbiome and how the gut microbiome interacts with tea polyphenols. Importantly, studies were compiled on the impact of differently processed tea, which contains different polyphenol profiles, on the gut microbiota from in vivo animal feeding trials, in vitro human fecal fermentation experiments, and in vivo human feeding trials from 2004–2024. The results were discussed in terms of different tea types and how their impacts are related to or different from each other in these three study groups.

## 1. Introduction

Tea is a highly popular beverage, primarily due to its unique flavor and aroma as well as its perceived health benefits [1]. Tea can be categorized into four main types based on processing methods: green tea, oolong tea, black tea, and dark tea, with additional minor white and yellow tea types [2]. Black tea, in which tea leaves are fully fermented, is the most widely consumed type, accounting for 76–78% of the global tea production, followed by green tea (unfermented), which contributes 20–22% [3,4]. In addition, oolong tea (semi-fermented) and Pu-erh tea (a unique form of dark tea specific to China that is produced from fermented tea with additional microbial fermentation) are also gaining increasing popularity among consumers [3,5]. Tea leaves contain polyphenols, alkaloids, amino acids, polysaccharides, and some proteins and minerals [6]. Tea polyphenols are the predominant tea phytochemicals and are mainly flavonoids, which are a diverse group of phenolic compounds [7,8]. Among the flavonoids, catechin (flavanol) is the major class that includes (+)-catechin, (−)-epigallocatechin-3-gallate (EGCG), epicatechin-3-gallate (ECG), (−)-epicatechin (EC), and (−)-catechin gallate (CG), and all these are considered the main bioactive constituents in tea [9,10]. Tea catechins exhibit strong antioxidant and biochemical activities with potential for mitigating conditions such as obesity [11], diabetes [12], neurodegenerative diseases [13], cardiovascular diseases [14], and certain cancers [15].

The gastrointestinal or gut microbiota in humans is often referred to as the “forgotten organ”, constituting an ecosystem composed of approximately 300–500 different bacterial species and totaling nearly 2 million genes [16,17]. Notably, the number of bacteria in the gut is estimated to be about 10 trillion, which is 10 times higher than the number of human cells, and the total number of genes in the microbiota far exceeds the human genome [16,17]. In the last two decades, it has come to be appreciated that the gut microbiota plays a crucial role in both human health and disease. Numerous studies have demonstrated the pathogenic role of gut dysbiosis in various diseases, including neurodegenerative diseases [18], obesity [19], diabetes [20], cardiovascular diseases [21], liver diseases [20], cancer [22], and multiple sclerosis [23]. The significant interaction between the gut microbial community and the host is based on the collective participation in mutual metabolic processes of food components between the host and microbes and the effect on the host immune system [16]. Dominated by the *Firmicutes*, *Bacteroidetes*, *Actinobacteria*, and *Proteobacteria* bacteria groups, the gut microbiota impacts drug metabolism and regulates digestion by processing nutrients, bile acids, and fibers. Bacterial fermentation of dietary fibers produces short-chain fatty acids (SCFAs) that are important energy sources for colonocytes and, when absorbed into the human host, modulate energy utilization, especially with regards to lipid synthesis and catabolism. Additionally, ‘friendly’ gut bacteria defend against invasive pathogens by competing for nutrients, colonizing space, and producing bacteriocins crucial to immune function [24].

Numerous studies have demonstrated that green, oolong, black, and Pu-erh teas have the capacity to modulate the composition of the gut microbiota, deriving from in vitro human fecal fermentation studies and in vivo animal and human feeding trials. This review tabulates and discusses these studies to contribute to a better understanding of the effect of differently processed tea on gut bacteria.

## 2. Tea

### 2.1. Bioactive Compounds in Tea

Polyphenols are a class of secondary metabolites widely distributed in plants, and these include tannins, ellagitannins, flavones, flavonols, isoflavones, flavanols, flavanones, and anthocyanins [25]. The polyphenol content in tea is predominately flavan-3-ols, but tea also contains caffeine and other pyrimidine derivatives in lesser quantities [26]. The phytochemical composition of tea varies significantly among different types of tea due to different post-harvest processing methods that impact the extent of chemical and enzymatic oxidation (referred to as fermentation) and microbial fermentation [27].

Catechins (flava-3-nols) are the primary polyphenols in green tea that include EGCG, ECG, EGC, and EC. These four species constitute about 90% of the total catechin content, and the potency of their antioxidant activity can be arranged in the following order: EGCG > EGC > ECG > EC [28,29]. In green tea, which is not fermented post-harvest, catechin content is the highest, ranging from 16–30%. In partially fermented oolong tea, catechin content varies between 8–20%, while in fully fermented black tea, catechin content is in the range of 3–10% [30]. In the case of Pu-erh tea, which is a post-fermented tea with an additional period of microbial fermentation, the catechin content was reduced to 0.027–5.52% [31,32]. However, black and Pu-er teas contain exotic oxidized tea products not found in the less oxidized teas [31]. 

Fermentation is a crucial step in the processing of tea, and it significantly influences the polyphenol content and profile of tea leaves, hence their flavors and aroma. Polyphenol oxidase, a tea enzyme that is released when tea leaves are harvested or crushed, drives the enzymatic fermentation of catechins and other polyphenols in tea leaves. It is a heat-sensitive enzyme; thus, post-harvest processing of the tea leaves involving heat treatment to terminate polyphenol oxidase activity differentiates the different tea types [33]. Green tea, with leaves treated with heat straight after harvest, retains the highest catechin content, while black tea undergoes full fermentation, resulting in a much lower catechin content [31]. During fermentation, catechins in tea leaves undergo oxidation and polymerization, forming orange-hued theaflavins, which can be further chemically transformed into red-hued thearubigins and dark-brown-hued theabrownins [34]. In the case of oolong tea, oxidation of the catechins during fermentation can be controlled by adjusting the degree of heat treatment, resulting in large variations in the phytochemical profiles, hence the oolong tea types, some of which resemble green tea and others black tea in their catechin content [27]. In the production of Pu-erh tea, green tea leaves are sun-dried and then processed into Pu-erh tea through an additional extensive and prolonged microbial fermentation step, resulting in further transformation of the oxidized phytochemicals, some into larger while others into exotic phytochemicals [35]. 

Tea polysaccharide (TPS) stands out as another significant bioactive component in tea, offering a spectrum of health benefits. Comprising a protein-bound acidic polysaccharide, TPS features a unique structure where 2–10 monosaccharides are intricately linked by glycosidic bonds, containing 44.2% neutral sugar, 43.1% glyoxylate, and 3.5% protein [36]. Processing methods play a crucial role in shaping the content and composition of TPS. Green tea polysaccharides (GTPS) exhibit a neutral sugar content ranging from 36.06–38.71%, while black tea polysaccharides (BTPS) contain protein-bound counterparts [37,38]. The MW distribution of TPS also varies depending on the type of tea and the process. Specifically, the MW of GTPS ranged from 9.2–251.5 kDa, the MW of oolong tea polysaccharides (OTPS) fell within a narrower range, and the lowest MW was that of BTPS [39,40].

### 2.2. Biological Transformation and Utilization of Tea Polyphenols and Polysaccharides

The catechin structure contains a chroman moiety in the A and C rings and a catechol group (3,4-dihydroxybenzene) in the B ring, which is attached to C2 of the C-ring. The absence or presence of a gallate group on C3 of the C-ring and the various stereoisomeric forms around the chiral C2 and C3 of the C-ring form the various species of catechins. However, flavonoids in food mainly exist in glycosylated forms, and glycosylation affects their absorption. Some deglycosylation can occur by the action of intestinal lactase-phlorizin hydrolase (LPH) in the small intestine in releasing the aglycone, but this activity is limited [41]. Importantly, from a bioavailability perspective, catechins are less glycosylated than other flavonoids [25]. The degree of glycosylation of tea catechin can vary based on factors such as tea variety, growing conditions, and processing techniques. Generally, green tea tends to have a higher percentage of catechins that are glycosylated compared to black tea, which has less than 50%.

After tea polyphenols are ingested, they are absorbed to varying degrees in the small intestine based on their aglycone structure. Approximately 5–10% of polyphenols can be absorbed, with some of the glycosylated moiety picked up by the glucose transporter and the aglycone moiety entering the intestinal epithelial cells through passive diffusion [42]. An active efflux exists in epithelial cells, which contributes to their overall low bioavailability [43].

Once absorbed, some catechins undergo phase I and II biotransformation in the small intestinal epithelial cells (enterocytes), but all are eventually transported to the liver via the hepato-portal vein, where further phase I and II biotransformation takes place. Phase I reactions lead to structural changes in the parent compounds to increase their polarity and involve processes such as hydrolysis, oxidation, and reduction reactions [44]. Phase II reactions introduce chemical groups to the structure as a detoxification step and enhance their urinary excretion, which included acetylation, methylation, sulfation, and glucuronidation reactions [42,45]. These processes lead to the release of acetylated, methylated, sulfated, and glucuronidated catechin conjugates, as well as other metabolites and unmodified catechin in lesser quantity, from the liver into the systemic circulation [46,47]. 

Both in vitro and in vivo studies have elucidated the chemical degradation of catechins in the stomach and small intestine. Specifically, catechins such as EGCG, EGC, and ECG undergo significant chemical degradation under gastrointestinal digestive conditions, leading to the formation of various homo- and heterodimers [48]. Dimer formation involves catechin autoxidation, where catechins scavenge O_2_ to create reactive semiquinone intermediates that can undergo dimerization. During the degradation process, EGC produces residual EGC, GC, and homodimers, whereas ECG results in residual ECG and CG without forming homodimers. The degradation of EGCG and ECG produces EGCG homodimers and EGCG–ECG heterodimers. In contrast, the combined degradation of EGC and ECG only generates EGC homodimers. The reactivity of the catechin intermediates influences the formation of these dimers, impacting their bioavailability and biological activities [35].

**Figure 1:**
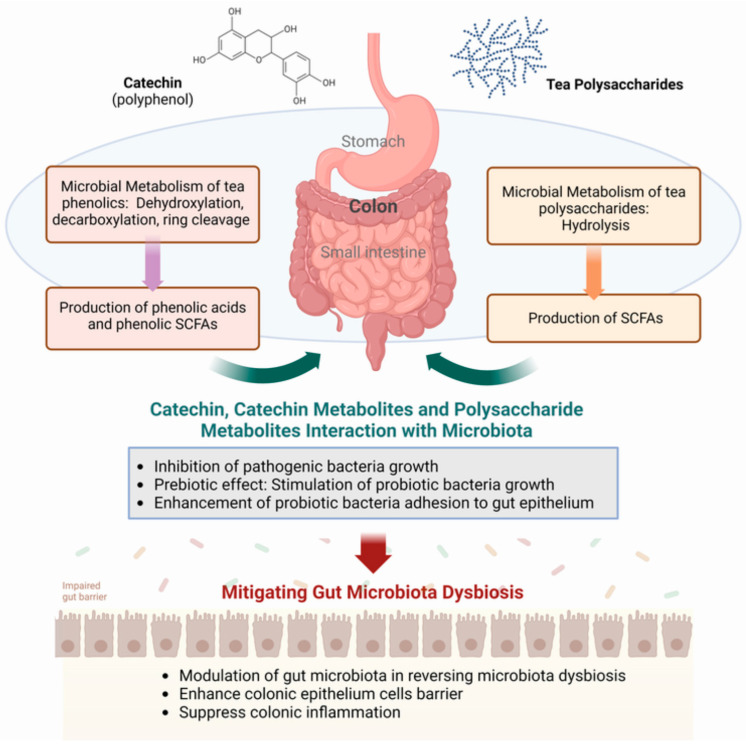
Modulation of the gut microbiota by tea polyphenols and polysaccharides. SCFAs, short-chain fatty acids. Figure generated using BioReader^®^.

Tea catechins escaping absorption and chemical degradation in the stomach and small intestine are presented to the colon, where the bacterial fermentation process takes place (Figure 1). Bacterial degradation capacity varies significantly among individuals, influenced by diet, host-microbiota interactions, and microbial interactions [49]. Microbial degradation involves specific microbial enzymes in generating a plethora of phenolic metabolite products, such as 3,4-dihydroxyphenylpropionic acid with 4-hydroxybenzoic acid, phloroglucinol, 3′,4′,5′-trihydroxyphenyl-γ-valerolactone, and 3′,4′-dihydroxyphenyl-γ-valerolactone from (+)-catechin and (−)-epigallocatechin ([35,50]). 

Complex polyphenols, especially those of condensed and oligomeric and polymeric structures such as ellagitannins, tannins, theaflavins, thearubigins, and theabrownins, are largely unchanged in the upper gut before being transported to the colon [51]. Such as the catechins, the colonic microbiota plays a crucial role in breaking down these complex polyphenols into absorbable, low-molecular-weight phenolic metabolites [52]. Here, they undergo microbial enzyme-promoted degradation that also includes C-ring cleavage and decarboxylation, dehydroxylation, and demethylation reactions [41]. These reactions lead to the formation of phenolic and hydroxy-cinnamic acids, and a significant amount of these are absorbed directly from the colon into the body.

In contrast to tea polyphenols, which undergo chemical and biochemical modifications in the upper and lower gut, all plant polysaccharides are carried to the colon unchanged, maintaining their structural integrity through gastric and intestinal digestions (Figure 1). In the colon, these polysaccharides undergo degradation facilitated by gut microbial enzymes that hydrolyze them into monosaccharides for use by the bacteria and other microorganisms that form the gut microbiota. The release of SCFAs into the colon from the bacterial processing of the sugars provided their physiological effects in the gut, similar to the bacterial processing of dietary fibers [53,54]. Thus, it can be observed that Fuzhuan Brick tea polysaccharides pass through the digestive tract with no change in molecular weight, monosaccharides, or reducing sugar content [55], and that tea polysaccharides undergo microbial breakdown mediated by the human fecal microbiota [56].

## 3. Human Gut Microbiota

The human intestinal mucosa has a surface area of 260–300 m^2^, providing an extensive habitat for gut microbiota [57], and contains trillions of microorganisms, forming a complex ecological community [58]. But the gut microbiota shows significant variations along the gastrointestinal tract. In the oral cavity, there are approximately 10^8^–10^10^ colony-forming units [CFU] of bacteria per gram of saliva. The bacterial count in the stomach is around 10^3^ CFU due to the strong and low acidic stomach environment, subsequently ranging from 10^2^–10^4^ CFU per gram of contents in the duodenum and jejunum, where the pH has elevated to near neutral, and ultimately increasing in the ileum (approximately 10^10^ CFU per gram of contents) and colon (ranging from 10^10^ to 10^12^ CFU per gram of contents), indicating a gradual increase in microbes along the digestive tract [59]. 

### 3.1. Diversity of Intestinal Flora

Based on culture-based studies, all healthy adults share a “core microbiota”, with most individuals harboring similar types of intestinal bacteria. However, DNA sequencing studies independent of cultivation have advanced the identification and understanding of microbial diversity, revealing that each person’s gut may contain over 1000 different species-level taxonomic units, contrary to the core concept [58]. The gut microbiota encompasses various microorganisms, including bacteria, yeast, protozoa, and viruses [60]. The major bacteria phyla in the gut microbiota include *Firmicutes*, *Bacteroidetes*, *Actinobacteria*, *Proteobacteria*, *Fusobacteria*, and *Verrucomicrobia*, with *Firmicutes* and *Bacteroidetes* constituting 90% of the total [61]. *Firmicutes* include over 200 different genera, such as *Lactobacillus*, *Bacillus*, *Clostridium*, *Enterococcus*, and *Ruminococcus*. Among these, *Clostridium* represents 95% of the *Firmicutes*. *Bacteroidetes* consist mainly of genera such as *Bacteroides* and *Prevotella*. *Actinobacteria*, represented by *Bifidobacterium*, are relatively fewer in number [62]. The composition of the gut microbiota in humans, while maintaining consistency in major components, exhibits significant variations in the relative proportions and specific species among individuals due to factors such as age, health status, host genetics, dietary patterns, medication use, and environmental factors [63].

However, the composition of microbial communities does not directly reveal their functions, necessitating research involving both cultured isolates and community DNA. Shotgun metagenomics involves sequencing total microbial community DNA and then matching these sequences with a database of known functional genes to screen for functions. While this method can identify genes involved in specific metabolic pathways, the predictions are based on database matching rather than actual analysis of mRNA, proteins, and metabolites [64].

With the development of high-throughput gene sequencing technologies, current methods for studying gut microbiota primarily involve two stages: first, bacterial gene sequencing based on 16S rRNA, and second, bioinformatic analysis [57]. Bacterial gene sequencing distinguishes different bacterial species by analyzing the DNA of 16S rRNA, a relatively small and highly conserved gene shared by all bacterial species, containing nine hypervariable regions sufficient for distinguishing different bacterial species [65,66]. The foundation of microbial phylogenetic classification lies in the genetic sequences of variable regions. To detect the DNA of any bacterial species, PCR primers can be designed to cover hypervariable regions, enabling the inclusion of variable regions in PCR amplification products. Identification of bacterial species or genera can be completed by sequencing the amplified PCR products and comparing them with known bacterial sequences in microbial databases [66,67]. Due to the extensive variability and abundant database information of 16S rRNA genes, it becomes a suitable target for widespread molecular analysis. Compared to traditional PCR, real-time PCR (RT-PCR) offers higher sensitivity and accuracy. RT-PCR allows real-time monitoring of DNA amplification through fluorescence intensity, eliminating the need for post-PCR detection techniques. Additionally, RT-PCR enables quantitative or semi-quantitative analysis, measuring the amount of DNA using the cycle threshold (Cq) value and indicating the cycle number when fluorescence intensity surpasses detectable levels [68]. However, the data obtained from sequencing is often extensive and messy, and bioinformatic analysis is necessary to clean up this data and identify bacterial taxa [57,68]. Moreover, statistical analysis of sequence data helps identify α diversity (species diversity within the same individual), beta diversity (species diversity between individuals), relative abundance, and other parameters related to the organism [57]. The α diversity analysis includes Chao 1, Simpson, and Shannon, where Chao 1 reflects community richness (number of different species), and Simpson and Shannon represent community diversity (taking into account the relative abundance of each species) [19].

### 3.2. Effect of Tea Polyphenols and Polysaccharides on Intestinal Microbiota

Diet significantly influences the composition of the gut microbiota, with components such as polyphenols increasingly recognized as playing a significant role alongside the well-studied effects of fats and dietary fibers (Figure 1) [51]. Polyphenols and their bacterial metabolic products affect the activity and composition of the gut microbiota, indicating a bidirectional relationship between them [47]. Thus, polyphenols promote the growth and establishment of probiotics such as *Bifidobacteriaceae* and *Lactobacillaceae*, which are recognized as beneficial bacteria in the human gut and have been approved for use as commercial probiotics. But polyphenols also inhibit the growth of certain pathogenic strains, such as *Clostridium difficile*, *Clostridium perfringens*, *Prevotella*, *Escherichia coli O157:H7*, and *Helicobacter pylori* [69]. Thus, when a person consumes a rich polyphenol-based diet, there is an increase in the abundance of *Lactobacillus*, *Bifidobacterium*, *Roseburia*, *Akkermansia*, *Faecalibacterium*, and *Prevotella*, while the ratio of *Enterococcus*, *Enterobacteriaceae*, and *Firmicutes* to *Bacteroidetes* significantly decreases [44]. This indicates that polyphenols contribute to the proliferation of beneficial bacterial populations while reducing the relative abundance of some harmful bacterial groups, thus exerting a positive impact on the gut microbiota composition that can be translated to better gut health.

Flavonols are the most common flavonoid compounds found in food and are also present in tea, albeit at a much lower level than catechins. Quercetin, a flavonol, can inhibit the growth of *Lactobacillus casei*, *Lactobacillus plantarum*, *Enterococcus faecalis*, *Bifidobacterium longum*, *Streptococcus suis*, and *Escherichia coli*, with minimum inhibitory concentrations ranging between 4–50 µg/mL [70]; Quercetin at 100 µg/mL significantly enhances the adhesion ability of *Lactobacillus acidophilus* to Caco-2 intestinal epithelial cells [71]. Improving the adhesion ability of probiotics, such as *L. acidophilus*, in the colon may enhance the gastrointestinal defense system by stimulating cytokine secretion, increasing mucin secretion, and improving intestinal tight junctions, among other mechanisms [63].

In batch-culture fermentation studies of human fecal bacteria, tea catechins reduce the growth of various pathogens such as *C. difficile*, *C. perfringens*, *S. pyogenes*, and *S. pneumoniae*, while also decreasing the growth of commensal anaerobic bacteria such as *C. sporogenes*. *Bifidobacterium* and *Lactobacillus* genera, as well as probiotics such as Lactobacillus, remain unaffected [72]. Additionally, (−)-epicatechin gallate, (+)-epicatechin gallate, and (−)-epicatechin promote the growth of beneficial *Bifidobacterium* and *Lactobacillus*/*Enterococcus* groups. In batch-culture fermentation of human fecal bacteria, these compounds inhibit the growth of *C. histolyticum* and Bacteroides-Clostridium groups [69]. Thus, catechins can significantly increase the production of SCFAs, including formic acid, acetic acid, propionic acid, and butyric acid, by increasing the number of these fiber-fermenting bacteria.

The impact of tea catechins on bacterial growth and metabolism depends on the polyphenol structure, dosage, and microbial strains. These catechins can interact with bacterial cell surfaces and inhibit bacterial enzyme activity, thereby affecting bacterial energy metabolism [73]. Studies have shown that ECG can make methicillin-resistant *Staphylococcus aureus* (MRSA) sensitive to β-lactam antibiotics, promote bacterial cell aggregation, and increase cell wall thickness [74]. Furthermore, catechins such as ECG and EGCG from green tea extracts have been found to significantly reduce the resistance of MRSA clinical isolates to β-lactam antibiotics such as methicillin [75]. 

It has been observed that EGCG enhances the in vitro resistance of alveolar macrophages to *Legionella pneumophila* infection through selective immunomodulation of cytokine formation [76]. Additionally, the impaired antibacterial and immune activities of alveolar macrophages caused by smoking can also be restored by EGCG treatment. In vitro studies have demonstrated that tea polyphenolic compounds, including EGCG, GCG, and EGCG3”Me, significantly inhibit the growth of *Bacteroides-Prevotella*, *Clostridium histolyticum*, and *Eubacterium-Clostridium* groups while having a lesser effect on the growth of *Bifidobacterium* and *Lactobacillus*/*Enterococcus* groups [77]. Moreover, a study using Chinese oolong tea revealed that EGCG, GCG, and EGCG promoted the growth of *Bifidobacterium* and *Lactobacillus*/*Enterococcus* groups while inhibiting the growth of *Bacteroides-Prevotella*, *Clostridium histolyticum*, and *Eubacterium-Clostridium* groups [69].

Tea polysaccharides also have a regulatory effect on the intestinal microbiota, mainly by acting as a probiotic and generating SCFA bacterial metabolites, thereby promoting the growth and proliferation of certain microbes and altering the composition of the gut microbiota (Figure 1) [78]. Thus, tea polysaccharides enrich *Prevotella*, and *Bacteroidetes* have been recognized as beneficial bacterial contributors to gut health [79,80]. It was shown that green tea polysaccharide conjugates (GTPC) increase the abundance of beneficial bacteria while decreasing the abundance of harmful bacteria [81]. In the sodium dextran sulfate-induced enteritis mouse model, tea polysaccharides were found to promote the growth of *Mycobacterium anisopliae*, resulting in enhancing intestinal epithelial barrier function and inhibiting intestinal and systemic inflammation. Acidic tea polysaccharides obtained from green tea were also found to exhibit an anti-adhesion effect against a range of pathogenic bacteria, namely *Staphylococcus aureus*, *Propionibacterium acnes*, and *Helicobacter pylori* [82]. Several animal experiments have substantiated the capacity of tea polysaccharides to effectively mitigate disruptions in the gut microbiota in hyperlipidemic rats. These interventions demonstrate a reduction in the proportion of thick-walled bacteria and Bacteroidetes, coupled with an elevation in the relative abundance of proficient SCFA producers, such as *Lachnospira* sp., *Victivallis*, and *Rossella* spp. [83]. This orchestrated microbial shift contributes to the amelioration of imbalanced glucose-lipid metabolism in rats.

Importantly, analogous effects have been observed in the human body, underscoring the translational relevance of these findings from rats to humans [84]. Fuzhuan Brick tea polysaccharide intervention also promoted the proliferation of beneficial microbiota (e.g., *Lactobacillus* and *Ackermannia*) with a concurrent increase in SCFA production, especially butyrate, in the cecum [85,86]. Moreover, tea polysaccharides demonstrated a modulatory impact on tryptophan metabolism within the gut. This modulation encompassed the generation of indole derivatives through microbial catabolism, subsequently activating the immune system. Ultimately, these processes had discernible effects on bolstering the integrity of the intestinal barrier [87].

### 3.3. Metabolic Influence of the Gut Microbiota

The gut microbiota produces SCFA through the fermentation of soluble dietary fibers and, to a lesser extent, insoluble fibers, with acetate (C2), propionate (C3), and butyrate (C4) being the primary SCFAs with an average ratio of approximately 60:20:20 [88]. SCFAs play crucial roles in providing a lumen-reducing environment, maintaining gut barrier function, participating in energy metabolism, regulating immune cell development, and exhibiting anti-inflammatory effects [89]. Among the SCFAs, butyrate serves as the main energy source for colonic epithelial cells (colonocytes), while acetate enters the liver via the portal vein and undergoes oxidation, primarily utilized by hepatic cells.

Different bacterial groups in the gut are responsible for the production of different SCFAs. For example, *Clostridium* and *Butyrivibrio* genera are major butyric acid producers, while *Bacteroides* and *Prevotella* genera are predominantly acetic acid producers [88]. Interconversion of the SFFAs by bacteria exists, with approximately 24% of acetate being converted to butyrate [90]. The importance of SCFAs in maintaining gut homeostasis and the intestinal environment is evident not only in their role in preserving gut barrier function but also in biochemical pathways such as regulating oxygen levels in the intestines, promoting colonocyte mitochondrial β-oxidation, and activating PPARγ [89]. Additionally, SCFAs produced in the gut, such as acetate and propionate, play a vital role in inducing colonic Treg cells, a specific subset of regulatory T cells that are found predominantly in the colon and are involved in immune responses and maintaining tolerance to self-antigens and harmless foreign antigens, thus suppressing intestinal inflammation [91,92,93].

The gut microbiota also has a significant impact on host lipid metabolism. Hepatocytes synthesize cholic acid and chenodeoxycholic acid, which, when combined with glycine or taurine, form water-soluble bile salts that are involved in the fat digestion process [94]. Intestinal bacteria convert primary bile salts into secondary bile salts, which are absorbed and circulated back to the liver via the hepatic-enteric recycling process. Bile acids not only emulsify and facilitate the digestion and absorption of dietary lipids but also serve as ligands for bile acid receptors, such as TGR5 and FXR, participating in the regulation of energy, cholesterol metabolism, and bile acid transporter gene expression [88,95]. Additionally, the gut microbiota synthesizes sphingolipids and regulates genes involved in sphingolipid biosynthesis. For example, the α-galactosyl ceramide produced by *Bacteroides fragilis* can inhibit the activation of natural killer T cells [96]. Moreover, the gut microbiota catalyzes the metabolism of unsaturated fatty acids using hydration and dehydration catalysis enzymes, producing metabolites such as hydroxy fatty acids [97]. The generation of these metabolites is associated with the regulation of physiological processes such as obesity and glucose metabolism. As an example of fatty acid receptors, the lack of FXR has been shown to be beneficial for overweight or obese individuals, resulting in reduced adipose tissue mass and weight gain, along with improvements in glucose homeostasis [98]. Additionally, TGR5 is another fatty acid receptor activated by binding with secondary bile acids such as deoxycholic acid and lithocholic acid. Upon activation, TGR5 induces the release of the glucagon-like peptide-1 (GLP-1) hormone from intestinal enteroendocrine L cells. GLP-1 has anti-diabetic properties, improving glucose metabolism by enhancing insulin secretion. Therefore, the activation of TGR5 plays a positive role in improving glucose tolerance in obese mice with diabetes [99,100].

## 4. Interaction of Tea Polyphenols with the Gut Microbiota

### 4.1. Literature Search Method

**Figure 2:**
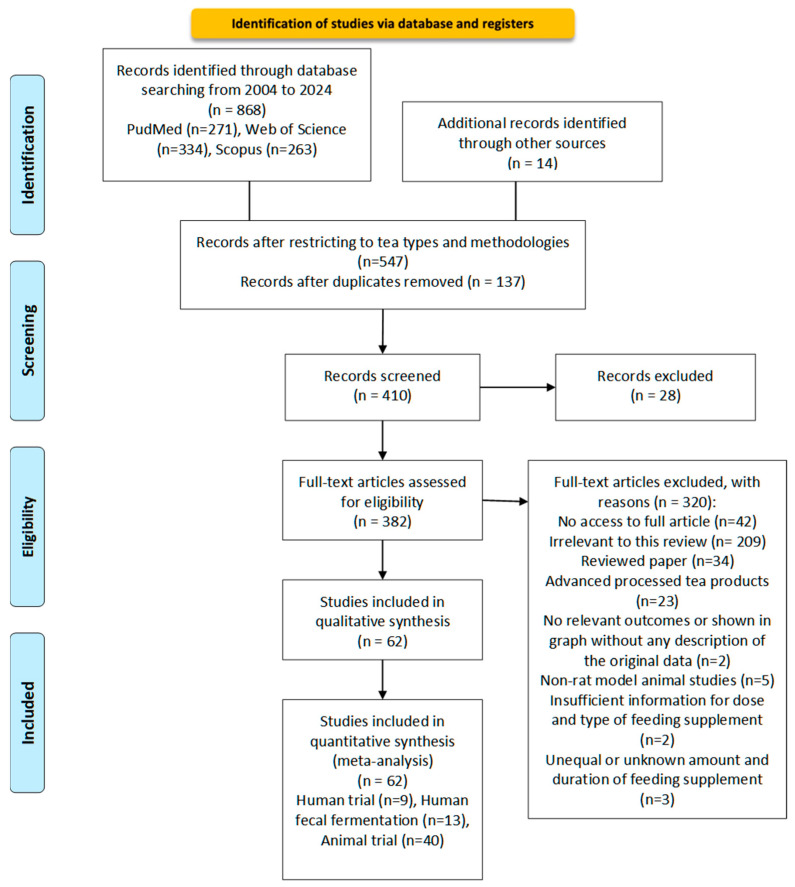
Flow diagram according to Transparent Reporting of Systematic Reviews (PRISMA statement). Study selection process for the meta-analysis of the effect of different processed teas on gut microbiota from 2004 to 2024 [101].

The literature search was conducted following the Preferred Reporting Items for Systematic Reviews and Meta-Analyses (PRISMA) guidelines [101]. Figure 2 presents the search strategy and search criteria based on the PRISMA flowchart.

#### 4.1.1. Search Strategy

Literature published in English between 2004–2024 was searched across three specialized databases (PubMed, Web of Science, and Scopus) according to the PRISMA guidelines. Search terms included (“Tea and gut bacteria AND/OR ‘Green’ or ‘Black’ or ‘Oolong’ or ‘Pu-erh tea’”) and (“in vitro” or “in vivo”) and (“Animal trial or Human trial”). Additional relevant studies were identified by manually searching the reference lists of selected studies and using Google Scholar. The searches were conducted between January and March 2024.

#### 4.1.2. Data Collection and Sorting

The literature search process located 868 journal articles across all databases from the years 2004–2024. The results were imported into EndNote X9 for compilation. Duplicates were identified and manually removed using the software.

#### 4.1.3. Eligibility Criteria

Selected studies were limited to animal and human research published entirely in English in peer-reviewed journals. The inclusion criteria were as follows: (1) were animal studies based on rat model in which rats were treated with tea or tea extracts; (2) were in vitro studies of fermentation experiments using human gut microbiota models, and fecal matter from healthy volunteer sources; (3) were randomized clinical studies in which humans were treated with tea or tea extract interventions; (4) clearly specified doses and durations; (5) the primary outcomes of interest included changes in gut flora (composition changes in gut microbiota(alpha diversity, and abundance, etc.); (6) secondary outcomes of interest included metabolic biomarkers (e.g., bile acids, SCFAs) and their associated metabolic effects, as well as the positive or negative health effects of changes in gut flora; (7) studies reporting outcomes related to the effects of tea on gut flora were included even if these were not their primary focus.

Studies were excluded if they: (1) were irrelevant to this review; (2) were review papers; (3) used advanced processed tea products as treatment instead of tea broth or tea extracts; (4) lacked relevant outcomes or presented data only in graphical form without descriptive statistics; (5) were non-rat model animal studies; (6) provided insufficient information regarding dose and type of feeding supplement; (7) included unequal or unknown amounts and durations of feeding supplements.

#### 4.1.4. Data Extraction

The data for each article are summarized in Table 1, Table 2 and Table 3, encompassing: general information (authors, year), statistical characteristics of subjects (species, number, age range, sex, health status, weight), intervention specifics (form, source, dose, duration), study design, and primary findings.

#### 4.1.5. Data Measures and Analysis

This analysis concentrates on the modulation of gut microbiota composition by tea and its extracts, evaluating metrics such as alpha (α) and beta (β) diversity, species richness, and evenness. Significant alterations in key bacterial taxa and their functional capacities are also examined. Key outcome indicators include microbial population identification and quantification via 16S rRNA gene sequencing and F/B ratios as markers of gut homeostasis. 

### 4.2. Rodent Feeding Trials

In a comprehensive review of studies on the effects of green tea, oolong tea, black tea, and Pu-erh tea on gut microbiota, distinct patterns and nuances emerge across various rodent feeding trials represented by rats and mice (Table 1 and Supplementary Table S1). The administration of tea samples was conducted in various forms, from extracts to infusions and decaffeinated formulations, with doses ranging from 0.05–2%. The microbial analytical techniques were notably consistent across studies, utilizing high-throughput sequencing techniques such as Illumina MiSeq or 16S rRNA gene sequencing for microbial analysis. Additionally, metabolomics approaches, such as using GC-MS or LC/MS for identifying metabolites, were commonly employed to evaluate metabolic changes induced by tea consumption. Various rodent species were employed, including Sprague-Dawley rats, C57BL/6J mice, and albino hairless mice, offering a broad spectrum of rodent species, strains, and health conditions for the studies.

The studies collectively show that green tea polyphenol (GTE) has a significant impact on rodent gut microbiota, with changes in gut microbiota composition, diversity, and abundance being common main findings. Among the major findings across studies, alterations in gut microbiota composition were consistently reported. An increased abundance of beneficial bacteria, such as the *Bacteroidetes* and *Lactobacillus*, and decreased levels of potentially harmful bacteria, such as *Firmicutes*, *Clostridium*, or *Turicibacter*, were shared outcomes. Several studies provided unique insights into discriminating factors affected by GTE, such as the family *ParaPrevotellaceae* [102], changes in the relative abundance of *Bacteroidetes* and *Firmicutes* [103], and associations with specific microbial species [104]. Furthermore, the studies consistently demonstrated an enhanced production of SCFAs, known for their positive effects on gut health. The association between green tea consumption and beneficial effects on body weight, glucose metabolism, and insulin resistance was another recurring theme, particularly evident in the rats and mice subjected to high-fat diets or induced obesity [105].

However, nuanced differences and species-specific responses emerged across the studies. Dosage effects on microbiota diversity were indicated in some studies [106], where increasing doses of green tea polyphenols resulted in a reduction in microbial diversity. On the contrary, others did not explicitly establish a linear relationship between dosage and outcomes, suggesting a more intricate interplay between polyphenols and microbes and the impact of phenolic and fiber SCFA metabolites [107]. Species-specific responses were evident, with variations observed in different rats, mouse species, or strains [108], emphasizing the importance of considering the specificities of the gut-microbiota interaction. Additionally, some studies highlighted the differential impact on specific bacterial families, such as *ParaPrevotellaceae*, or genera, such as *Akkermansia* and *Turicibacter*, indicating nuanced responses within the microbiota composition [107]. Moreover, certain studies explored the impact of green tea on disease models, such as the LPS-induced colitis model, with findings suggesting potential ameliorative effects on symptoms and dysbiosis, adding a layer of complexity to the understanding of green tea’s influence on gut health [109].

Oolong tea, with its partially oxidized, thus more diverse polyphenol profile, presented intriguing findings regarding its impact on gut microbiota. The studies collectively focus on exploring the impact of oolong tea polyphenols (OTP) on gut microbiota across various contexts, encompassing circadian rhythm disruption, high-fat diet-induced obesity, high-salt diet-induced hypertension, disrupted uric acid metabolism, and alcohol-induced liver disease. Although each investigation delves into different scenarios, there are discernible similarities in key findings and methodologies. Thus, it was observed that the positive effects of purified OTP on the colon included up-regulation of the relative abundance of the ‘good’ bacteria *Akkermansia* and *Muribaculum* while down-regulation of *Desulfovibrio*, affirming the positive impact of tea polyphenols on the gut microbiota [110].

In the context of the circadian rhythm, alteration may lead to changes in the microbial population, subsequently impacting various metabolic pathways, including the synthesis and transportation of microbial metabolites in the gut, such as neurotransmitters, SCFA, and bile acids. These changes could potentially exert negative effects on maintaining the metabolic homeostasis of the organism [111]. A study uses a circadian rhythm-disrupted mouse model to investigate the impact of OTP water extracts on the gut microbiota [112]. Continuous dark exposure resulted in unclear rhythmic changes in the mouse gut microbiota, while OTP significantly improved the suppression of circadian rhythmic changes at the phylum level induced by continuous darkness. This intervention restored the rhythmic changes in the abundance of *Firmicutes*, *Bacteroidetes*, *Verrucomicrobia*, and *Proteobacteria*; specifically, there was a decrease in *Firmicutes* and a relative increase in *Firmicutes*/*Bacteroidete* ratio. The increase in this bacteria ratio in the gut microbiota has been linked to positive impacts on health, including obesity, metabolic health, and reduced gut inflammation [113]. A study of a human gut ecosystem model by transplanting fresh adult fecal microbiota into germ-free mice also obtained similar results from an alteration of the circadian rhythm [114].

It can be observed that high-fat diet-induced obesity produces consistent patterns in microbial alterations induced by oolong tea [115,116]. These include increased *Bacteroidetes* abundance, decreased *Firmicutes*, and a resulting reduction in the *Firmicutes*/*Bacteroidetes* ratio. Notable reductions in specific bacterial genera, such as *Lachnospira*, are reported, a bacterial group involved in the breakdown of complex carbohydrates such as cellulose and hemicellulose. Additionally, a decrease in *Firmicutes* and an increase in *Bacteroidetes* occur after oolong tea treatment, specifically promoting the growth of beneficial bacteria such as the *Bifidobacterium* and *Lactobacillus* genera [117,118]. An experiment with a high-salt diet-induced hypertension animal model also shows the regulatory effects of oolong tea on the gut microbiota in mice [119]. This study indicated that oolong tea supplementation significantly decreases *Firmicutes* and increases *Bacteroidetes*, thereby reducing the *Firmicutes*/*Bacteroides* ratio. Moreover, specific enrichment for genera including *Allobaculum*, *ParaPrevotella*, *Oscillospira*, *Bifidobacterium*, and *Ruminococcus* is observed at the genus level, with beneficial effects on health largely due to the roles these bacteria play in maintaining gut homeostasis and supporting various physiological functions.

In addition to healthy rodents, diseased rats were also studied. Relative abundance of *Firmicutes* decreased and *Bacteroidota* increased significantly in disrupted uric acid metabolism in a rat study [120]. After oolong water extract treatment, the relative abundances were reversed, leading to a significant improvement in the *Firmicutes*/*Bacteroidota* ratio, signifying a shift in microbial composition. The regulatory effects of oolong tea extracts on the gut microbiota in alcohol-induced liver disease mice were also studied [121]. The findings show that oolong tea intervention restores intestinal flora richness and inhibits alcohol-induced reduction of *Verrucomicrobia* and *Actinomycetes*. The formal is a bacterial group that specializes in degrading mucin, contributing to the maintenance of the integrity of the gut barrier, and supporting gut health, while the latter is a bacterial group that plays a role in improving immune function and reducing inflammation. It also decreases the relative abundance of *Bacteroidetes* and significantly increases that of *Firmicutes*, positively increasing the *Firmicutes*/*Bacteroidota* ratio.

Oolong tea polyphenols consistently demonstrated regulatory effects on gut microbiota across various studies, despite differences in interventions and treatment durations. Black tea exhibits similarities to oolong tea in influencing gut microbiota, as supported by existing evidence, even though the tea is characterized by a higher level of tea catechin oxidation. In contrast to green tea and oolong tea, however, black tea exhibits a more intricate and diverse impact on gut microbial composition, diversity, and abundance. This complexity is underscored by the contradictory results observed in various experimental settings, highlighting the nuanced nature of the effect of black tea’s polyphenol composition arising from tea processing on influencing the gut microbiota.

It was observed that there was a significant reduction in a subcluster of the fiber fermenter *Clostridiales* within the *Clostridium cluster XIVa* in healthy mice following black tea ingestion [122]. This was accompanied by a slight increase in the median abundance of bifidobacteria. In contrast, other studies showed an increase in alpha diversity without significant changes in *Proteobacteria* but a notable increase in *Firmicutes* and a decrease in *Bacteroidetes*, thereby elevating the *Firmicutes*/*Bacteroidetes* ratio [123]. Interestingly, a consistent decrease in the relative abundance of the *Lactobacillus* genus was noted, indicating a complex impact on the gut microbiota from the more oxidized and structurally complex black tea catechins such as theaflavin.

Studies investigating the effects of black tea on mice fed high-fat diets reveal conflicting results as well. Significant alterations in the intestinal microbiota of mice fed a high-fat diet were observed, characterized by an increase in *Firmicutes* and a decrease in *Bacteroidetes*, leading to an elevated *Firmicutes*/*Bacteroidetes* ratio [124]. However, feed supplementation with black tea extracts did not significantly impact this ratio in this study, suggesting that black tea had a lesser effect on modulating the gut microbiota compared to oolong tea. By contrast, it was observed that the rise of *Lactobacilli* induced by a high-fat diet is inhibited in mice, showcasing the ability of specific black tea to ameliorate the elevated abundance of *Duberella* by exerting a significant inhibitory effect on heterogeneous bacteria [19]. Another study noted changes in the relative abundance of specific bacterial classes and OTUs induced by a high-fat diet in mice [125]. Black tea supplementation impacted the abundance of 56 OTUs, with an increase in 18 and a decrease in 38 units, with notable increases in *E. coprostanoligenes*, *L. gasseri*, and *B. S24-7* and a decrease in *Roseburia* spp. and *E. coli*. Other studies found a relatively smaller effect on alpha and beta diversity in mice, with minimal influence on the *Firmicutes*/*Bacteroidetes* ratio [126]. Continuing, in studies adopting a low-fat, high-sugar diet to induce changes in rat physiology, supplementation with black tea diets led to significant modifications at the genus level [127]. The diets were associated with a noteworthy increase in the relative proportions of *Parabacteroides*, *Bacteroides*, and *Prevotella*. Conversely, several genera from the *Firmicutes phylum*, including *Roseburia*, *Lactobacillus*, *Blautia*, *Anaerostipes*, *Shuttleworthia*, *Bryantella*, *Lactococcus*, and *Acetitomaculum*, experienced a significant decrease. This highlights the significant impact of black tea on gut bacteria diversity.

The influence of intestinal microbiota on alcoholic fatty liver disease in mice has also been studied, with findings showing that treatment with black tea water extracts had minimal effects on gut microbiota richness and diversity [121]. Nonetheless, black tea significantly decreased the relative abundance of *E. coli* and increased the relative abundance of *Verrucomicrobia, Para Bacteroidetes,* and *Actinobacteria*. Regarding high uric acid levels, it was demonstrated that both black and green tea treatments significantly decreased the relative abundance of *Bacteroidetes* [120]. Additionally, the *Firmicutes*/*Bacteroidota* ratio also significantly decreased in shifting the microbial composition. Black and green tea treatments also resulted in a reduction in the relative abundance of *Bacteroidetes* and *E. coli*, impacting fiber fermentation. Another study exploring the effects of black tea on aging-related cognitive dysfunction found that black tea’s theaflavin treatment led to an increase in the relative abundances of *Actinobacteria* and the *Firmicutes*/*Bacteroidetes* ratio, which have positive and negative health impacts, respectively, on the gut [128]. The same study also shows that theaflavin effectively restored the altered relative abundances of *Bifidobacterium*, *Clostridium*, *Bacteroides*, *Bacteroidetes*, *Roseburia*, and *Lachnospiraceae* at the genus level in D-galactose-induced aging mice, indicating a positive impact on the *Firmicutes*/*Bacteroidetes* ratio and alpha diversity suggestive of a beneficial effect.

With Pu-erh tea, another layer of complexity is introduced due to the distinct processing methods involving extensive microbial fermentation of tea polyphenols post-oxidation that resulted in the formation of new and complex structures such as thearubigins and theabrownins [35]. Notably, certain studies draw comparisons and delve into the nuanced similarities and differences between raw and cooked Pu-erh tea, further contributing to the depth of understanding. With raw Pu-erh tea, leaves are typically sun-dried right after picking and then undergo a natural microbial fermentation and aging process over a range of years. With ripe (or cooked) Pu-erh tea, leaves are piled together in a warm and humid environment to accelerate microbial fermentation over a period of weeks to months. It can be shown that both raw and ripened Pu-erh tea theabrownin reversed the altered abundance of specific bacterial groups induced by a high-fat diet, increasing beneficial bacteria such as *Bacteroides* and *Akkermansia* while reducing commensal and harmful bacteria groups such as *Lactobacillus* and Streptococcus [129,130]. Specifically, ripened Pu-erh tea showed greater potential for adjusting dysbiosis and normalizing the Firmicutes/Bacteroidetes ratio compared to raw Pu-erh tea.

It was observed that both raw and ripe Pu-erh tea administration affected core functional genes and enzymes in the cecal microbiota, impacting carbon metabolism and genetic information processing [130]. Enriched enzymes from *Akkermansia muciniphila* in response to Pu-erh tea administration were identified, providing insights into the functional changes induced by Pu-erh tea extracts. This bacterium plays a crucial role in maintaining gut barrier function because it feeds on mucin, promoting its degradation and turnover and maintaining the integrity of the gut barrier. Delving into the effects of ripe Pu-erh tea on gut microbiota composition, a study identified alterations in the abundance of specific bacteria in response to Pu-erh tea water extract supplementation [131]. Similarly, another study observed that Pu-erh tea aqueous extracts increased the biodiversity of cecal bacterial communities in rats fed a high-fat and high-sugar diet [132]. Notably, Pu-erh tea administration resulted in increased relative abundances of *Firmicutes*, particularly the genus *Lactobacillus*, and *Actinobacteria*, including the genus *Bifidobacterium*, indicating effects on promoting beneficial bacteria growth.

It can be shown that the mice ingestion of Pu-erh tea infusion reduced the populations of specific bacterial groups, including OTUs in the *Lactobacillus*, *Bacillus*, *Enterococcus*, *Lactococcus*, *Streptococcus*, and *Leuconostoc genera* [133]. These reductions imply a regulatory effect on these bacteria, potentially influencing various metabolic processes within the gut. The effect of ripened Pu-erh tea on female C57BL/6 mice reveals specific alterations in the gut microbiota [134]. In this study, Pu-erh tea treatment reversed the DSS-induced increase in the relative abundance of the fungus *Aspergillus* and the decrease in the thick-walled phylum. Additionally, this study also shows that Pu-erh tea treatment significantly increased the abundance of specific bacterial groups, including *Enterobacteriaceae* and *Helicobacteraceae*. This was supported by another investigation that shows the effect of Pu-erh tea polyphenols (aqueous extraction) in increasing the abundance of specific bacterial groups, including *Fusobacterium*/*Clostridium globosum* group, *F. prausnitzii*, *A. muciniphila*, and *Bifidobacterium* spp., pointing to a positive impact on the microbial community [135].

In summary, the exploration of green tea, oolong tea, black tea, and Pu-erh tea in in vivo rodent studies reveals a complex interplay between tea consumption and modulation of the gut microbiota. The unique characteristics of each tea type, ranging from their specific effects on bacterial taxa to their diverse metabolic implications, provide a foundation for further specific research into the potential health benefits of tea consumption. Green tea consistently reveals positive impacts on gut health in rodents, fostering beneficial bacteria and metabolic improvements. The increased presence of beneficial bacteria, such as *Bacteroidetes* and *Lactobacillus*, coupled with reduced levels of potentially harmful bacteria, such as *Clostridium*, suggests a positive impact on gut health. The association with enhanced SCFA production further highlights potential benefits, extending to the impact of body weight, glucose metabolism, and insulin resistance. Oolong tea demonstrates reliable regulatory effects on the gut microbiota in diverse scenarios, showcasing potential therapeutic applications. From circadian rhythm disruption to high-fat diet-induced obesity and disease models, oolong tea consistently demonstrates positive influences. An increased abundance of beneficial bacteria decreased the *Firmicutes*/*Bacteroidetes* ratio, a decrease associated with a lower or lower level of obesity, inflammation, metabolic syndrome, and autoimmune diseases. Black tea, characterized by higher levels of catechin oxidation, presents a complex influence with varying outcomes across studies. In addition to the effect on microbial abundance in increasing microbial diversity, the ability to modulate gut health in disease models, such as alcoholic fatty liver disease and disrupted uric acid metabolism, further adds layers to its potential therapeutic applications. Pu-erh tea, with its unique processing methods for raw and cooked variants, introduces complexity to gut microbiota research. Both raw and cooked Pu-erh tea extracts demonstrate regulatory effects, influencing specific bacterial groups and core functional genes. The ability of Pu-erh tea to reverse altered bacterial abundance induced by high-fat diets and its positive impact on circadian rhythm disruption underline its potential therapeutic applications.

In synthesizing the findings across green tea, oolong tea, black tea, and Pu-erh tea regarding their effects on gut microbiota, it becomes evident that the diverse outcomes observed in rodent experiments may stem from inherent biological variations, differences in study design, and varying metabolic responses. However, the controlled conditions of the rodent studies, involving concentrated forms of tea extracts and specific dosages, may not accurately reflect the diversity of human consumption patterns and lifestyle factors. It is essential to be aware that the translation of these results to the human context is nuanced and influenced by factors such as individual human gut microbiota variability, genetics, diet, and lifestyle. The differences between animal experiments and human clinical trials may arise from the inherent dissimilarities in gut microbiota compositions and metabolic processes between species. Navigating this intricate landscape of tea and its impact on gut health, human clinical trials become paramount for a comprehensive understanding relevant to human nutrition. By bridging this gap between animal experiments and human feeding trials, there is a chance to unravel the full scope of tea’s potential benefits for the gut microbiota.

**Table 1:** Animals Feeding trails. Methodology and Major Findings are presented in Supplementary Table S1.

Tea Type	Reference	Subjects	Treatment
Green Tea	[102]	Female ovariectomized Sprague-Dawley rats (six months old, Harlan Laboratories, Indianapolis, IN)	0.5%, or 1.5% of (g/mL) green tea polyphenol extracts (decaffeinated) each day
	[106]	Specific pathogen-free C57BL/6J mice	0.05%, 0.2% and 0.8% (*w*/*w*) green tea polyphenols (commercial), added to the drinking water each day
	[127]	C57BL/6J mice	Decaffeinated 0.25% (*g*/*g*) Green tea polyphenol (ethanol extracts) added in diet every day
	[105]	Seven-week-old male C57/BL6 mice	400 mg/kg green tea extracts (50% ethanol extracts dissolved in water) every day
	[136]	Five-week-old female db/db and wild-type mice	AIN-93M diet with 1% and 2% (*w*/*w*) dried green tea water extracts and green tea leaf powder (pulverized tea leaves) every day
	[137]	Male C57BL/6J mice (n = 50; 5 weeks old)	2% (*w*/*w*) purified green tea extracts, 0.3% (*w*/*w*) EGCG (HF + EGCG), or 0.3% (*w*/*w*) CAT (HF + CAT)) every day
	[138]	Female albino hairless mice (Skh:HR-1, 6–8 weeks old)	1% (*w*/*w*) green tea extracts (commercial) in diet each day
	[139]	C57BL/6 female mice (20 ± 2 g, 7–8 weeks)	5 mg green tea water extract powder/kg bodyweight each day
	[140]	Germ-free C57BL/6J mice (6 weeks old)	0.1% (*w*/*w*) Green Tea Polyphenols (water extracts) each day
Oolong Tea	[114]	Young adult (6-week-old) male C57BL/6J mice	0.1% (*w*/*w*) purified Oolong Tea Polyphenols (water extracts) each day
	[115]	young adult (6-week-old) male C57BL/6J mice	2% (*w*/*v*) purified Oolong tea polyphenols (water extracts) each day
	[121]	The 8-week-old C57BL/6J male mice	200 mg/kg body weight Black tea powder (water extracts, concentrated, and dried) each day
	[112]	Sterile male mice C57BL/6J	0.1% (*w*/*w*) Oolong tea polyphenols (water extracts) each day
	[110]	Six-week-old male C57BL/6J mice (6–8 weeks old, 23 ± 2 g)	200 mg/kg body weight oolong tea polyphenols (purified) each day
	[116]	6-week-old male C57BL/6J mice	0.1% (*w*/*w*) oolong tea ground powder and purified EGCG3″Me each day
	[118]	6-week-old male C57BL/6J mice	0.1% *w*/*w* purified oolong tea polyphenols (water extracts) each day
	[117]	6-week-old male C57BL/6J mice	0.1% (*w*/*w*) oolong tea ground powder and purified EGCG3″Me each day
	[119]	Twenty-four 8-week-old cleaning Wistar male rats	500 mg/kg oolong tea water extract added to drinking water each day
	[120]	Seventy-two male specific-pathogen-free grade Kunming mice, with a weight of 35–40 g at 6 weeks of age	800 mg/kg·d oolong tea water extract, administered by gavage once a day for two consecutive weeks
Black Tea	[126]	ICR mice, aged 8 weeks and weighing 22 ± 2 g (32M and 32F)	25 mg/mL black tea water brewed each day
	[121]	The 8-week-old C57BL/6J male mice	200 mg/kg b.w. black tea extract powder (dried water extracts) each day
	[19]	The 8-week-old C57BL/6J male mice	200 mg/kg b.w. black tea extract powder (dried water extracts)
	[125]	6-week C57BL/6J male mice	760 mg/Kg. Average energy intake. Purified black tea water extracts
	[127]	48 male C57BL/6J mice at 6–7 weeks of age (body weight: 16–18 g)	320 mg/kg body weight ethanol-water black tea extracts each day
	[124]	48 C57BL/6 mice (male, 6 weeks)	2.0% black tea ground powder [*w*/*w*] each day
	[123]	Male SD rats (6 weeks of age, n = 14)	1.5 g/kg body weight black tea powder (suspended in distilled water) per day
	[120]	Seventy-two male specific-pathogen-free grade Kunming mice, with a weight of 35–40 g at 6 weeks of age	800 mg/kg·d black tea water extracts (freeze-dried powder)
	[122]	Four-week-old male Wistar rats (n = 21)	10 g/kg Black tea extracts (ethanol-aqueous freeze-dried powder) each day
	[128]	Eight-week-old male ICR mice weighing 35–40 g	50 mg kg/1 Black tea theaflavins (commercial) every day
Pu-erh tea	[133]	3-week-old C57BL/6J male mice	3 mg/mL instant Pu-erh tea (PT) water infusion (450 mg/kg/day)
	[131]	8-week-old male C57BL/6N mice	0.4% and 1% (*w*/*v*) Ripened Pu-erh tea water extracts each day
	[132]	Male Wistar rats fed a high-fat and high-sugar diet (HFSD) to induce obesity	0.15-g/kg and 0.4-g/kg body weight PTE (aqueous extracts) each day
	[141]	Male Sprague-Dawley rats (weighing 180–220 g)	Pu-erh tea water extracts 0.16 g/mL, 15 mL/kg/day
	[129]	42 male C57BL/6 mice (6 weeks of age, body weight 20.4 ± 1.0 g)	600 mg/kg/d and 300 mg/kg/d raw or ripened Pu-erh tea water extracts
	[135]	Six-week-old C57BL/6J male mice	Daily doses of 750 mg/kg of body weight Pu-erh tea (aqueous extraction) each day
	[134]	Female C57BL/6 mice (8 weeks old; 17–20 g)	3%, 6% and 9% (*w*/*w*) Ripened Pu-erh tea water extracts each day
	[142]	24 mice (12 males and 12 females)	0.15 mL Pu-erh tea water extract each day
	[130]	Fifty C57BL/6J male mice	400 mg/kg raw Pu-erh tea theabrownin (R-TB) or ripened Pu-erh tea theabrownin (F-TB) (purified) each day
	[143]	male C57BL/6J mice	0.25% (*w*/*v*) Pu-erh tea (water extracts, freeze-dried powder) each day
	[144]	Pathogen-free C57BL/6 mice (8 weeks of age, weighing 20 ± 2 g)	0.1 or 0.4% (1 or 4 g/L, *w*/*v*) Pu-erh tea freeze-dried water extracts each day

### 4.3. Human Fecal Fermentation 

The amalgamated studies considered here collectively enhance our nuanced comprehension of the intricate interplay between green tea consumption and the human gut microbiota. Note that adding tea directly to fecal fermentation would include all the polyphenols and other chemical components in tea that would have otherwise been absorbed and/or degraded while passing through the upper gut before reaching the colon. The results of the human fecal fermentation studies discussed here are presented in Table 2 and Supplementary Table S2.

While each study provides a unique perspective on this interaction, a common thread emerges: green tea exerts discernible effects on the composition and functionality of the gut microbial community. An investigation has been conducted into the impact of purified GTE powder on individuals with metabolic syndrome using fresh fecal material from subjects fermented in a Human Colonic Model system that included distinct tea feeding phases of subjects, including a 14-day equilibrium period, a 7-day pre-treatment phase, a 14-day treatment phase, and a 7-day washout phase [145]. Metagenomic and metabolomic analyses in this study reveal significant shifts in gut microbiota composition at the genus level, with specific bacterial groups responding differentially across colon compartments. Notably, supplementation with GTE results in an augmented production of free fatty acids by gut microbes. In another study, a human colon microbiota system was used to simulate meal intake and scrutinize the repercussions of GTE supplementation [146]. The result reveals a decrease in *Firmicutes* and a modest decline in *Bacteroidetes*, coupled with an increased abundance of SCFA-producing bacteria. These findings underscore the potential of green tea to modulate the human gut microbiota in a manner conducive to better health.

In a study with mixed Lahpet, a traditional Burmese dish made from fermented or pickled green tea leaves, and human fecal samples, 16S rRNA gene sequencing discerned differential effects on bacterial abundance at the phylum level and alterations in SCFA production [147]. The intricate relationship between ethanol-extracted green tea polyphenol (GTP) and gut microbiota is further elucidated in another study that focused on gut bacteria cultures [148]. The result reveals the inhibitory effects of GTP on various bacterial species, underscoring the nuanced and species-specific responses within the human microbial community, similar to the effects on rodents. Noteworthy is the biotransformation of the polyphenols into compounds with enhanced antioxidant activity, which would contribute to ameliorating the colonic oxidative stress induced by IBD.

The dynamic nature of catechin degradation and microbiota responses was shown in a study that employed fecal materials from healthy adults [149]. This study reveals a promotion of beneficial bacteria, such as *Bacteroides* and *Bifidobacterium*, alongside the inhibition of some potentially harmful bacteria. This adaptive response of the microbiota over time reinforces the dynamic nature of the human gut ecosystem when exposed to tea catechins. Fermentation of purified green tea polyphenols with human fecal samples also sheds light on their impact on gut bacteria and SCFA production [150]. The results indicated enhanced proliferation of beneficial bacteria, such as *Bifidobacterium* and *Lactobacillus*/*Enterococcus*, alongside the inhibition of several potentially harmful bacteria, contributing to an overall positive modulation of the gut microbial community.

All the in vitro studies mentioned employed models with fecal samples or gut microbiota cultures and commonly utilized advanced analytical techniques, including sequencing, metabolomics, and statistical tools, to investigate the impact of GTE on the gut microbiota within a simulated environment. Shared findings across these studies encompass alterations in microbiota composition and increased production of SCFAs. Notably, there were consistent observations of heightened levels of the bacterial groups *Escherichia* coli and *Klebsiella* and of SCFAs following GTE treatment. Conversely, a reduction in the abundance of *Bacteroides* and *Clostridium* was consistently noted. These collective outcomes underscore a consistent trend in the modulation of gut microbial communities by GTE across diverse experimental setups, providing a robust basis for understanding its effects on the human gut microbiota. While the studies collectively reveal the modulatory effects of green tea on the gut microbiota, several distinctions exist. Divergent subject characteristics, experimental designs, and the specific GTE or compounds used contribute to the variability in observed outcomes. Nevertheless, the consensus on the capacity of green tea to induce shifts in microbiota composition and foster the growth of beneficial bacterial populations underscores its potential as a modulator of human gut health.

There is much less research on the use of oolong tea in human fecal fermentation studies, which may be due to the fact that oolong teas are very diverse and sit between green and black tea in terms of the degree of catechin oxidation. In one study, the effects of specific catechins in oolong tea on the intestinal microbiota through in vitro fermentation of healthy human feces were examined [69]. Their work indicates that EGCG, GCG, and EGCG demonstrated stimulatory effects on the proliferation of the fiber fermenter *Bifidobacterium*, *Lactobacillus*, and *Enterococcus* groups while simultaneously exhibiting inhibitory effects on the growth of the *Bacteroids Prevotella* and *Eubacterium-Clostridium* groups. Furthermore, the total concentrations of SCFAs in fermentation cultures containing EGCG, GCG, and EGCG were notably higher compared to the control group without catechin addition, correlating to the increase in the fiber fermenters, *Bifidobacterium* and *Lactobacillus*. These observations were supported by another study with oolong tea in their human fecal fermentation setup [150].

In vitro studies on black tea have predominantly employed anaerobic fermentation setups to simulate the anaerobic metabolic environment of the human gut. Through a comprehensive analysis of these studies, black tea exhibits a range of positive regulatory effects on the intestinal microbiota. As early as 2013, there was research employing the Human Simulator of the Microbial Ecosystem (SHIME) to mimic the human gastrointestinal environment [151]. It was observed that BTE significantly reduced the relative abundance of *Actinobacteria* and *Firmicutes* in response to both single and continuous doses. *Bacteroidetes*, on the other hand, were not significantly affected. At the phylum level, the most substantial change was an increase in *Proteobacteria*. Additionally, the overall family-level abundances of *Ruminococcaceae* and *Lachnospiraceae* decreased with a higher black tea dosage. Black tea also stimulates the proliferation of *Klebsiella*, *Enterococcus*, and *Akkermansia* while reducing the relative abundances of *Bifidobacterium*, *Bacillus coccidioides*, *Anaerobic cocci*, and *Victivallis* in the same study. These observations were also supported by another study that investigated the in vitro modulatory effects of green tea, oolong tea polyphenols (water extracts), and black tea polyphenols (water extracts) on the human intestinal microbiota [150]. 

A recent study utilizing an in vitro fermentation model found that black tea led to significant alterations in the relative abundances of bacterial taxa at the class, order, and family levels [152]. Notably, *Fusobacteria* and *Bacteroidetes* increased, which is consistent with a previous report that black tea had no significant increase in the relative abundance of *Bacteroidetes* [151]. It was also observed that black tea can cause an increase in the relative abundance of *Fusobacterila* and *Bacteroidia*, while *Gammaproteobacteria*, *Negativicutes*, and *Clostridia* decreased [124]. These trends were confirmed at the genus and family levels, with certain groups, such as Fusobacteriaceae, *Bacteroidaceae*, and *Burkholderiaceae*, increasing and others, such as *Lachnospiraceae*, *Tannerellaceae*, *Acidaminococcaceae*, and *Enterobacteriaceae*, decreasing. Additionally, a study on the effect of theaflavin, a major oxidized product in black tea, on fecal bacteria through in vitro anaerobic human gut microbial fermentation shows similar modulatory effects on the gut microbiota [153]. At the genus level, a decrease in *Bacteroidetes* and an increase in *Ruminococcaceae* were observed. Treaflavin significantly increased the relative abundance of unidentified *Ruminococcaceae*, *Clostridium trichophyton*, *Flavonifractor*, *Eubacterium*, *Ruminococcaceae*, *Clostridium trichophyton*, and *Blautiae* species.

In summary, research on black tea consistently demonstrates a positive regulatory effect on the gut microbiota. Consumption of black tea has been associated with the stimulation of certain bacterial groups, including *Klebsiella, Enterococcus,* and *Akkermansia*. In contrast, black tea has been shown to decrease the relative abundance of some major fiber fertilizers, such as *Bifidobacterium*, *Clostridium*, *Anaerostipes*, and *Victivallis*. While the overall impact of black tea on the gut microbiota is consistently positive, the variations in specific bacterial responses and taxonomic changes underscore the complexity of the interaction between black tea and the human gut microbiota.

The main findings from human fecal fermentation studies on green tea and oolong tea share some similarities. Both green tea and oolong tea have a stimulatory effect on beneficial fiber-fermenting bacteria such as *Bifidobacterium* and *Lactobacillus*/*Enterococcus* groups. Secondly, both teas induce changes in the composition of the gut microbiota, affecting the relative abundance of different bacterial groups. Additionally, they can increase the production of SCFAs related to the increase in fiber fermenters, which can positively impact gut health. Moreover, both teas exhibit inhibitory effects on specific bacterial groups, such as *Clostridium perfringens*, *Clostridium difficile*, and the *Clostridiales* group, some of which are protein fermenters. While there are differences in the tea phytochemical profile between green and oolong tea, the observed microbial responses seem similar in terms of the effect on fiber and protein fermenters, but with distinction. The specific fiber fermenter bacterial groups affected differ between the two teas, with oolong tea primarily influencing genera such as *Bacteroides* and *Clostridium*, while green tea predominantly affects *Bifidobacterium* and *Lactobacillus*/*Enterococcus* genera. In summary, while green tea and oolong tea share a common theme of positively influencing the gut microbiota, differences in fermentation levels and unique compositions may result in specific impacts and targeted bacterial groups between the two teas.

Black tea and green tea share common positive effects on the human gut microbiota, again demonstrating their regulatory influence on bacterial abundance and beneficial bacteria. Both teas stimulate the proliferation of beneficial bacterial groups, such as *Bifidobacterium* and *Lactobacillus/Enterococcus*. Additionally, they inhibit the growth of specific bacterial groups, although the affected bacterial groups may differ between the two teas. For example, black tea reduces the relative abundance of *Bifidobacterium, Anaerostipes, Anaerotruncus,* and *Victivallis,* while green tea only exhibits inhibitory effects on some of these bacterial species. It was shown that black tea shows no significant impact on *Bacteroides* [151], while green tea increases the abundance of *Bacteroides* over time [149]. Research on green tea typically emphasizes green tea polyphenols’ inhibitory effects on specific bacterial species, whereas research on black tea highlights its impact on the overall microbiota profile [148] Furthermore, both black tea and green tea contribute to an increase in the production of SCFAs, potentially providing health benefits to the gut. While they share common positive effects, subtle differences in the gut microbiota may stem from differences in their polyphenol compositions. Overall, these findings underscore the potential of black tea and green tea as regulators of human gut health through their influence on the gut microbiota.

In conclusion, all four types of tea can impact the composition and abundance of the gut microbiota in human fecal fermentation, exerting certain effects on the microbiota profile. They have shown the ability to increase the production of SCFAs, contributing positively to gut health. Additionally, these teas exhibit specific effects on certain bacterial groups; they may stimulate beneficial bacteria and inhibit pathogenic bacteria. However, due to differences in fermentation levels, with green tea being unfermented, oolong tea partially fermented, black tea fully fermented, and Pu-erh tea with additional microbial fermentation, they vary significantly in chemical composition and molecular structure [154], and these play out in differences in their effects on the human gut microbiota.

**Table 2:** Human fecal fermentation studies. Methodology and Major Findings are presented in Supplementary Table S2.

Tea Type	Reference	Subjects	Treatment
Green Tea	[145]	Fresh fecal samples were donated by three adults with metabolic syndrome (two males and one female)	2% (*w*/*v*) GTE freeze-dried powder each day
	[146]	Human Colon Microbiota (from human fecal samples of two healthy volunteers)	0.67 mg/mL GTE freeze-dried powder (boiling water extracts) each day
	[147]	Fresh fecal collected from five healthy adults who had not received antibiotics or pre/probiotics in the preceding 3 months	1% (*w*/*v*) freeze-dried Laphet (fermented green tea) ground powder each day
	[148]	Gut microbiota culture (*Actinobacteria*, *Bacteroidetes*, *Firmicutes*, *Proteobacteria*, and *Verrucomicrobia*)	0.5 mg/mL GTE dried green tea powder (70% ethanol extracts) each day
	[146]	Fecal materials from four healthy volunteers (three males and one female, 24–38 years)	0.1 mmol/L catechin (EC, ECG, EGC, and EGCG) (purified) each day
	[155]	Fecal from ten healthy adult volunteers (six women and four men) aged between 33 and 70 years (average, 47 years)	Participants drank 1000 mL of green tea brew each day
	[150]	Fecal samples were obtained from 6 healthy volunteers (3 females and 3 males, aged 25–30 years) without recent antibiotic treatment or gastrointestinal disease	1% (*w*/*v*) freeze-dried green tea polyphenols (purified) each day
Oolong Tea	[150]	Fecal samples were obtained from 6 healthy volunteers (3 females and 3 males, aged 25–30 years) without recent antibiotic treatment or gastrointestinal disease	1% (*w*/*v*) freeze-dried oolong tea polyphenols (purified)
	[69]	Fecal samples were obtained from three healthy volunteers (one female and two males, ages 25–30)	100 mg/L oolong tea EGCG, GCG, EGCG3”Me (purified) each day
Black Tea	[150]	Fecal samples were obtained from 6 healthy volunteers (3 females and 3 males, aged 25–30 years) without recent antibiotic treatment or gastrointestinal disease	1% (*w*/*v*) freeze-dried black tea polyphenols (purified) each day
	[152]	Two healthy volunteers (one male and one female, aged 22–28 years) who had never had gastrointestinal disease and had not taken antibiotics in the past 3 months	2% (*w*/*v*) black tea polyphenols (70% ethanol extracts) each day
	[151]	Identical fecal sample from a healthy human volunteer	1000 mg black tea polyphenols each day
	[153]	Fresh human feces were collected from 8 healthy Chinese volunteers (18–24 years old; 4 males and 4 females)	0.1 mg/mL black tea theaflavins (purified) each day

### 4.4. Human Feeding Trials

Human feeding trials provide the most relevant data for efficacy in terms of health effects, as they account for the absorption and degradation of tea phytochemicals, including tea polyphenols, as they pass through the upper digestive tract prior to their delivery to the colon, affecting the gut microbiota. The number of feeding trials is, however, limited, probably due to the complexity and cost of conducting such experiments. Table 3 and Supplementary Table S3 tabulate the few studies gathered on the effect of tea in human feeding trials.

In the realm of human trial studies on green tea, the recruited adults exhibited diverse characteristics, encompassing varying age ranges, BMI statuses, and overall health conditions. Lower alpha diversity was observed in overweight subjects at baseline, with no significant differences noted in the response to the daily intake of GTE commercial capsules between normal-weight and overweight participants [156]. However, other studies show substantial changes in overall microbial diversity following green tea intake [157,158]. Taxonomic changes, specifically alterations in microbial families (*Lachinospiraceae*, *Ruminococcaceae*, and *Erysipelotrichaceae*) and genera (*Roseburia*, *Faecalibacterium*, and *Bifidobacterium*), were demonstrated with green tea intake [157]. These contrasting results could in part be due to differences in the degree of catechin degradation and individual microbiota variability in the test subjects. 

Exploring the relationship between green tea intake and gut microbiota, specific microbial species linked to green tea intake and their potential role in mediating glucose levels can be identified [104]. In addition, on the impact of catechins and EGCG intake on metabolic endotoxemia, gut barrier integrity, and the gut microbiota in individuals with metabolic syndrome (MetS), some consistency in taxonomic changes and associations with metabolic markers was observed [159]. The evidence collectively points towards a potential role of green tea consumption in modulating the gut microbiota in vivo, particularly in metabolically diseased populations.

There are only a limited number of in vivo human studies exploring the impact of OTP intake on the gut microbiota. In a study involving 30 healthy adults aged 20 to 50 years, a 3-week intervention with a water brew from 2.5 g oolong tea leaves significantly restored the alpha diversity of the gut microbiota in overweight individuals, characterized by increased observed species, Shannon Index, and Accumulated Cyclone Energy Index [160]. However, this restorative effect was not observed in individuals with a normal body weight. Taxonomic composition changes induced by oolong tea treatment varied based on participants’ BMI, with overweight participants exhibiting a significant reduction in *Megamonas* and an enrichment of *Bacteroidetes* and *Prevotella*. In contrast, normal-weight participants did not show a significant intervention effect on the relative abundance of *Bacteroidetes* and *Prevotella*. These results differ from animal and in vitro human fecal fermentation experiments, reflecting variations in dosage, study duration, subject characteristics, and bacterial analysis methods, emphasizing the challenges of in vitro experimental outcomes in human subjects.

With regards to Pu-erh tea, structural changes in the fecal microbial communities of healthy male volunteers were observed, noting a decrease in *Bacillus* and *Clostridium* abundance with Pu-erh tea consumption [134]. Pu-erh tea extract has also been shown to provide protective effects against alcohol-induced damage in mice, the restoration of gut microbiota diversity, and the *Firmicutes*/*Bacteroidetes* ratio [144]. These findings underscore Pu-erh tea’s potential in modulating gut microbial balance in whole animals and in promoting health in humans. By investigating the impact of exclusive green tea consumption on fecal microbiota over a ten-day period, a study underscores individual-specific changes in the composition of bifidobacteria in response to green tea consumption using DNA analysis techniques [155]. 

In these human feeding trials, both green tea and oolong tea exhibit both similarities and differences in their impact on the gut microbiota. Both teas stimulate beneficial bacterial growth and increase SCFA production, thus contributing to overall gut health. However, the specific taxonomic changes induced by each tea type differ. Green tea studies report alterations in microbial families and genera, highlighting its potential role in influencing gut microbiota composition. On the other hand, oolong tea studies reveal a restorative effect on gut microbiota alpha diversity in overweight individuals.

Shifting the focus to black tea, a 2004 randomized, double-blind, crossover study comparing black tea to a placebo found no statistically significant changes in the abundance of specific bacteria when analyzed using fluorescent in-situ hybridization with specific probes [161]. However, while black tea did not affect the specific bacterial groups analyzed, it reduced the overall abundance of bacteria detected by a universal bacterial probe. A recent clinical trial with a randomized, single-blind, parallel-group, and placebo-controlled design revealed that black tea-fed subjects experienced a significant rise in the abundance of *Prevotella,* accompanied by a simultaneous decrease in fecal acetic acid concentration [162]. This effect was more pronounced in individuals with lower salivary secretory IgA (SIgA) levels. As the trial progressed, the group consuming black tea displayed more evident alterations in the overall bacterial composition, including total, specifically butyrate-producing, bacteria that included *Prevotella*. The abundance of *Prevotella* is associated with immune modulation and digestive and metabolic health. Methodological differences, such as the use of fluorescence in situ hybridization (FISH) [161], could contribute to differing results.

When comparing green tea and black tea, significant differences emerge in their effects on the human gut microbiota in feeding trials. In green tea studies, which encompass diverse participant characteristics, they demonstrate potential taxonomic changes and associations with metabolic markers. However, the evidence suggests that the impact of green tea on the gut microbiota might be more pronounced in metabolically diseased populations than in healthy individuals on a normal diet. By contrast, black tea studies, particularly in a recent clinical trial [162], highlight significant changes in the abundance of specific bacteria, such as *Prevotella*, and alterations in overall bacterial composition. Collectively, these human feeding experiments shed light on the impact of green tea, oolong tea, and black tea on the gut microbiota in a whole-body context. While both green tea and oolong tea have demonstrated effects on microbial diversity and composition in certain studies, black tea appears to exert a relatively minor influence. In the realm of green tea studies, a consistent trend towards stimulating beneficial bacterial populations has emerged. However, the impact on overall microbial diversity varies, and specific microbial changes differ across studies. Concurrently, the association between green tea and metabolic effects has garnered attention, particularly in individuals with metabolic diseases. For black tea, studies consistently reveal a positive regulatory effect on the intestinal microbiota. Nevertheless, the specific microbial changes and directions vary, indicating the intricate nature of the different tea effects. Human feed trials with Pu-erh tea have yet to be conducted, indicating this area of research is still in its infancy.

**Table 3:** Human feeding trials. Methodology and Major Findings are presented in Supplementary Table S1.

Tea Type	Reference	Subjects	Treatment
Green Tea	[157]	Fecal samples from adult volunteers aged between 27 and 46 years. (Healthy, normal weight (BMI 18–24 kg m^2^), or overweight/obese (BMI > 24 kg m^2^)	400 g 7.5 g-Tea/L-Water green tea liquid each day
	[163]	58 Healthy Caucasians aged 18–50 years, either normal weight (BMI 18–25 kg/m^2^) or overweight/obese (BMI > 25 kg/m^2^), non-smokers, weight stable, and not on certain medications or specific medical conditions Clinical trial ID: NCT01556321	GTE capsules (>0.56 g/day EGCG + 0.28 ∼ 0.45 g/day caffeine)
	[164]	187 healthy postmenopausal women aged 50–60 years from the Minneapolis-St. Paul metropolitan area with a body mass index (BMI) between 19.3 and 36 kg/m^2^ and stable weight were enrolled	GTE Catechin Complex (Corban Complex GTB; Investigational New Drug #103,431) Intake: 1315 ± 115.0 mg catechins, including 843.0 ± 44.0 mg EGCG (capsules) each day
	[104]	A total of 85 participants (65.9% male; mean age: 43.3 years) without T2DM were included	The mean (SD) intakes of total green tea were 443 (417) mL/day, respectively. The median intakes of catechins and EGCG were 67.8 (23.2–150.1) and 9.5 (0.9–26.9) mg/day (green tea brew)
	[159]	20 individuals with MetS and 20 age- and gender-matched healthy persons. Clinical trial ID: NCT03973996	GTE (1g containing 890 mg of total catechins) confections each day
Black Tea	[162]	Fecal from 72 Healthy males or females between 20 and 59 years of age. Clinical trial ID: UMIN000038168	3 cups of Black tea brew (polymerized polyphenols, 76.2 mg) each day
	[161]	Fecal samples from a human volunteer	1 cup of black tea brew (made from dry tea powder) each day (There is no prescribed time or dosage for drinking black tea)
Oolong Tea	[160]	Fecal samples were obtained from 28 healthy adults, ranging in age from 20 to 50 years, whose weight had been stable for at least 3 years. Clinical trial ID: NKUIRB2022085	500 mL 5 mg/mL oolong tea water brew (Total phenolic content: 636.17 ± 8.54 μg GAE/mL) each day
Pu-erh tea	[165]	Thirteen healthy male volunteers, 24–32 years	300 mL Pu-erh tea brew (powder) (50 mg/kg) each day

## 5. Comparison among Different Teas in the Same Study

Comparing different teas in the same study would provide a more reliable comparison than with results from different studies, as it would eliminate sample and experimental variability. Sun and Chen [150], for example, established an in vitro anaerobic fermentation model using fresh feces from healthy volunteers to study the bacterial community during the fermentation of different tea polyphenols. During the fermentation process, the number of *Bifidobacteria* gradually increased and remained stable in the media containing water extracts of green tea, oolong tea, and black tea polyphenols. All the tea had a promoting effect on *Bifidobacteria*, with OTP and BTP showing better effects than GTP. For the *Lactobacillus*/*Enterococcus* species, tea polyphenols also had a positive impact on their proliferation. There were no significant differences among the three teas in inhibiting the proliferation of *Bacteroides-Prevotella* and *Clostridium histolytica*.

In in vivo experiments intervening with green and black tea in diets in healthy mice, green tea significantly reduced the relative abundance of *Prevotella* (*p* < 0.05), *C. subcluster XIVa* (*p* < 0.05), and *Clostridium* cluster XI (*p* < 0.001) while increasing *Lactobacillales* (*p* < 0.001) [122]. However, the BT also significantly reduced *C. subcluster XIVa* (*p* < 0.01) and *C. cluster XI* (*p* < 0.05). Both interventions showed a slight increase in the median values of *Bifidobacterium* abundance, though they did not reach statistical significance. In studies of the species composition of the fecal microbiota in mice treated with water-extracted GTE, OTE, and BTE in feeding experiments, all three teas demonstrated positive influences on gut microbiota composition in the context of hyperuricemia, a condition characterized by elevated levels of uric acid in the blood linked to obesity [120]. The *Firmicutes*/*Bacteroidota* ratio, indicative of microbiota balance, decreased after tea treatment, suggesting potential therapeutic benefits for Hyperuricemia. However, subtle differences were observed in the specific bacterial responses. GT treatment showed notable increases in beneficial bacteria such as *Ruminococcus* and *Lactobacillus*. BT treatment exhibited distinct modulations in bacteria related to uric acid metabolism. OT treatment, while sharing similarities with GT and BT, showcased its unique impact on specific bacterial taxa. Notably, the GT-treated mice had the highest microbial diversity, surpassing the effects of OT and BT.

Investigation of the impact of water-extracted GT and OT on the intestinal microbiota of rats on a high-salt diet shows that oolong tea supplementation in feeds reversed the observed abundances of specific bacterial taxa significantly, while GT intervention significantly altered the relative abundance of certain bacterial taxa in a different manner [119]. In addition, studies of the lipid-lowering effects of GT and BT in mice show that both teas increased the abundance of Proteobacteria, primarily due to the enrichment of Enterobacteriaceae [126]. Genera enriched by green tea were positively correlated with beneficial biochemical indices, while genera upregulated by black tea were associated with adverse lipid and metabolic parameters. 

In another study, the impact of decaffeinated green and black tea ethanol-water extracts on body weight gain in mice fed a high-fat diet was investigated, aiming to discern the role of gut microflora in the antihypertrophic effects of GT and BT [127]. Bacterial DNA sequencing in the mice unveiled significant alterations in cecum phyla composition. Mice subjected to a High Fat/High Sugar (HF/HS)-GTE and BTE diet resulted in a more substantial rise in *Bacteroidetes* and a reduction in *Firmicutes* and *Actinobacter* compared to mice subjected to a Low Fat/High Sugar (LF/HS) diet. At the genus level, positive correlations with body weight included *Blautia*, *Bryantella*, *Collinsella*, *Lactobacillus*, *Marvinbryantia*, and *Turicibacter*, while negative correlations were noted for Barnesiella and Parabacteroides. The HF/HS-GTP and BTP diets were linked to an increase in *Parabacteroides*, *Bacteroides*, and *Prevotella*, along with a decrease in various *Firmicutes* and *Actinobacter* genera. GTP consumption induced changes in *Clostridium* and *Coprococcus*, whereas BTP consumption led to an increase in *Oscillibacter*, *Anaerotruncus*, and Pseudobutyrivibrio. LF/HS diet mice exhibited an increase in *Bacteroides* and a decrease in *Blautia* and *Bryantella* compared to HF/HS diet mice.

Up until the present moment, there appear to be no human feeding trials comparing different types of tea within a single study. This is not surprising, given the cost and complexity of such trials, which would require many human subjects to yield meaningful results.

## 6. Conclusions

The alterations in intestinal microbiota composition were consistently demonstrated in most in vitro human fecal fermentation studies, with enhanced populations of beneficial bacteria and inhibition of harmful bacteria. These studies, conducted in controlled environments, offer valuable insights into the direct effects of tea polyphenols on the gut microbiota. However, they lack the complexity of the whole human digestive system, including systemic absorption and degradation, and, furthermore, do not account for interactions with dietary factors. Human clinical trials provide more nuanced and accurate insights on tea’s impact on gut health, though they often reveal variability in individual responses. Many of these trials focus on specific populations and yield macro-metabolic conclusions, such as minimal changes in overall microbial diversity or effects on glucose levels. Few studies report significant changes in bacterial abundance, suggesting that methodological factors may influence the results.

The intricate interplay between tea varieties and gut microbiota highlights the dynamic nature of these interactions. The differential modulation of microbial communities by green tea, black tea, oolong tea, and Pu-erh tea underscores the importance of considering specific tea types in understanding their potential health effects. Most studies from rodent feeding trials and from human fecal fermentation experiments and feeding trials show that tea has a positive impact on gut health by modulating the gut microbiota and that it can alleviate gut dysbiosis caused by a high-fat diet, hyperuricemia, and aging.

This review provides insight for future research, emphasizing the need to elucidate the specific pathways through which tea polyphenols and polysaccharides impact the gut microbiota. A focus should be placed on the molecular interactions between these bioactive compounds and microbial communities. Employing advanced metabolomic and metagenomic techniques will be crucial in mapping the metabolic transformations of tea-derived compounds within the gut, identifying the precise microbial enzymes involved, and characterizing the resulting metabolites. Such investigations would enhance our understanding of how different tea varieties modulate specific microbial populations and their metabolic outputs, including SCFAs and other bioactive compounds that are integral to gut health and systemic physiology.

Moreover, exploring the ‘synergistic’ effects of tea polyphenols and polysaccharides with other dietary components, such as fibers, prebiotics, and probiotics, could yield valuable insights into optimized dietary strategies for enhancing gut health. Further research on the influence of different brewing methods, tea processing techniques, and the bioavailability of tea compounds will be critical to maximizing the therapeutic potential of tea.

Long-term, large-scale human clinical trials are essential to validate findings from animal models and short-term studies, particularly within diverse populations that exhibit varying dietary habits and health statuses. These studies should aim to establish dose-response relationships, assess the effects of chronic tea consumption, and explore the potential of tea as a therapeutic intervention in gut-related diseases, such as inflammatory bowel disease, metabolic syndrome, and colorectal cancer.

## Data Availability

Data will be made available on request.

## Supplementary Materials

The following supporting information can be downloaded at: https://www.mdpi.com/article/10.3390/molecules29174020/s1, Supplementary Table S1: Animal Feeding Trails, Supplementary Table S2: Human Fecal Fertilization Studies, and Supplementary Table S3: Human Feeding Trails.
