# Supplementary material for: Effects of Differently Processed Tea on the Gut Microbiota
Source: Molecules. 2024 Aug 25;29(17):4020. doi: 10.3390/molecules29174020 (PMC11397556; doi:10.3390/molecules29174020)
Supplement: Supplementary file 1 [file molecules-29-04020-s001.zip › molecules-3148727-supplementary.pdf]

Effects of Differently Processed Tea on the Gut Microbiota

Zimo Zhao, Ruofan Chen and Ken Ng

Supplementary Tables

Supplementary Table S1: Animal feeing trails.

Tea Type	Reference	Subjects	Treatment	Methodology	Major findings
Green Tea	[102]	female ovariectomized Sprague-Dawley rats (six months old, Harlan Laboratories, Indianapolis, IN)	0.5%, or 1.5% (g/ml) green tea polyphenols extracts (decaffeinated) each day	Rats were randomly divided into six groups and fed a pelleted AIN-93M diet. They had ad libitum access to either regular distilled water (control) or distilled water mixed with 0.5% or 1.5% GTP (g/ml), prepared daily. The experiment lasted either 3 or 6 months. Animal weights were recorded weekly, and food and water consumption were monitored daily. Overnight fasting blood samples were collected for haematological and clinical chemistry analyses. Colon contents were stored at -80°C for microbial DNA extraction, followed by PCR amplification. SMC-seq libraries were prepared and sequenced using an Illumina NextSeq instrument.	Increasing doses of GTP in experimental rats reduced the biodiversity of intestinal communities, with samples from the 1.5% GTP group having significantly lower biodiversity indices.<br>At month 6, 5 *Clostridiales* and 2 *Erysipelotrichales* of *Firmicutes*, 1 *Bacteroidales* decreased with GTP doses, and 3 *Bacteroidales*, 1 *Clostridiales* (*Oscillospira*), 1 Desulfovibrionales of *Proteobacteria* increased. The phylum *Bacteroidetes* and its descendant phylotype, *Bacteroidia*, *Bacteroidales*, and family Bacteroidaceae, were all enriched with increasing GTP doses. One family *Peptostreptococcaceae* of *Firmicutes* decreased. Family *ParaPrevotellaceae* of *Bacteroidetes* was the only phylotype that was identified as discriminating between different dosing groups, which decreased with increasing GTP doses.
	[106]	Specific pathogen free C57BL/6J mice	0.05%, 0.2% and 0.8% (w/w) green tea polyphenols (commercial), added to the drinking water each day	Specific pathogen-free C57BL/6J mice were housed in controlled sterile environments. Mice were orally inoculated with human fecal suspensions and divided into five diet groups: normal diet (ND), high-fat diet (HFD), HFD with 0.05% tea polyphenols (TP), HFD with 0.2% TP, and HFD with 0.8% TP. Fecal samples were collected for 16S rRNA sequencing. After 8 weeks of TP administration, oral glucose tolerance tests were performed following a 12-hour fast. Fat and liver weights, as well as serum lipid levels, were measured post-sacrifice. Short-chain fatty acids in feces were quantified using GC-MS, and metabolic profiling was conducted with Chem-Station software.	The groups TPM, TPL, and TPH showed clear distinctions in relative abundance of *Bacteroidetes*. Predominant phyla were *Bacteroidetes*, *Firmicutes*, *Proteobacteria*, and *Actinobacteria*. Relative abundance of *Bacteroidetes* decreased in HFD but increased in TPM and TPH. *Firmicutes* showed the opposite trend, higher in HFD but lower in TPL and TPH.

[127]	C57BL/6J mice	Decaffeinated 0.25% (g/g) Green tea polyphenol (ethanol extracts) added in diet every day	Tea polyphenol extract was prepared from 500 g of tea leaves using 75% ethanol. After ethanol removal, the extract was purified, decaffeinated, and dried. In animal studies, 48 male C57BL/6J mice were divided into four diet groups: low-fat/high-sucrose, high-fat/high-sucrose, and high-fat/high-sucrose supplemented with green tea polyphenols (GTP) or black tea polyphenols (BTP). The extracts provided 0.25 g polyphenols per 100 g diet. After 4 weeks, mice were euthanized, and tissues were collected for analysis.	Sequencing of bacterial DNA in mice revealed notable changes in cecum phyla composition. The HF/HS-GTP diet led to a significant increase in *Bacteroidetes* and a decrease in *Firmicutes* and *Actinobacteria* compared to other diets. On the genus level, positive correlations with body weight included *Blautia*, *Bryantella*, *Collinsella*, *Lactobacillus*, *Marvinbryantia*, and *Turicibacter*, while negative correlations were observed for *Barnesiella* and *ParaBacteroides*. The HF/HS-GTP diets were associated with an increase in *ParaBacteroides*, *Bacteroides*, and *Prevotella*, and a decrease in several *Firmicutes* and *Actinobacteria* genera. GTP consumption induced changes in *Clostridium* and *Coprococcus*, while BTP consumption led to an increase in *Oscillibacter*, *Anaerotruncus*, and *Pseudobutyrivibrio*. GTP contribute to subject weight loss, which is associated with altered microbiota.
[105]	Seven-week-old male C57/BL6 mice	400 mg/kg Green tea extracts (50% ethanol extracted)(dissolved in water) every day	After a 1-week acclimatization, mice were randomly assigned to normal and high-fat diets for 12 weeks, with daily oral administration of green tea. Body weight and food intake were recorded weekly. Blood glucose levels were measured using the TheraSense Freestyle Meter kit. Stool samples were collected, and microbial DNA and EVs were extracted using the PowerSoil DNA isolation kit. The bacterial 16S rDNA V3/V4 region was amplified by PCR and analyzed with the MiSeq system.	green tea reduced glucose and insulin resistance in diet-induced obese mice (green tea did not affect glucose and insulin tolerance on a normal diet).<br>At the phylum level, HFD altered the composition of the gut microbiota, and the HFD-induced decrease in *Akkermansia* abundance was significantly reversed by GT. EGCG may be a major promoter of *Akkermansia* growth.
[136]	Five-week-old female db/db and wild-type mice	AIN-93M diet with 1% and 2% (w:w) dried green tea water extracts and green tea leaves powder (pulverized tea leaves) every day	Green tea powder (GTP) was prepared by pulverizing Japanese green tea leaves, and green tea extract (GTE) was extracted with hot water and spray dried. After a 5-day acclimatization, mice were randomized into 5 groups and fed test or control diets for 11 weeks. Fasting blood samples were collected on Days 14, 28, and 77, and blood glucose was measured with the Ascensia Contour meter. Serum insulin levels were measured using a Rat/Mice Insulin ELISA kit. Fresh fecal samples were collected daily, and genomic DNA was extracted with the QIAmp Power Fecal DNA kit. Food intake, body weight, and fasting glucose levels were analyzed using ANOVA and Tukey's test.	Decreased fasting blood glucose levels and fasting serum insulin water in mice with 1% GTE in the diet.<br>*Fusobacterium*, and *Trichoderma*, was boosted by 1% GTE and 2% GTP. 1% GTE and 2% GTP both promote *Lacotbacillus*, *Bifidobaterium*, *Parvibacter* and *Lachnospiraceae*.<br>Muribaculaceae, *Lachnospiraceae*, *RuminiClostridium*, *Oscillibacter*, *Clostridium*, *Roseburia* and *Ruminococcaceae* are reduced by 2% GTP while increased by 1% GTE.

[137]	Male C57BL/6J mice (n = 50; 5 weeks old)	2% (w:w) purified green tea extracts, 0.3% (w:w) EGCG (HF+EGCG) or 0.3% (w:w) CAT (HF+CAT)) every day	Male mice were fed various diets for 8 weeks, including a high-fat (HF) diet or the same HF diet supplemented with 2% GTE, 0.3% EGCG, or 0.3% CAT. Body mass, fecal samples, serum, and tissues were monitored and collected. Total RNA was extracted from tissues for RT-qPCR using a CFX384 instrument with an SYBR Green PCR kit. Gut microbiota analysis involved DNA extraction, 16S rRNA gene sequencing, diversity assessment via QIIME2, taxonomic classification using the Silva database, and functional predictions using PICRUSt2.	The HF diet significantly increased the *Firmicutes*:*Bacteroidetes* (F:B) ratio at the phylum level, which was mitigated by GTE and CAT supplementation, particularly in the HF+EGCG group.<br>At the order level, HF-induced changes in *Clostridiales* and Anaeroplasmatales were prevented by EGCG. *Coriobacteriales* increased in the HF+EGCG and HF+CAT groups. Mollicutes RF39, elevated in the HF group, was attenuated by GTE, EGCG, and CAT. Genus-level analysis demonstrated that HF feeding and tea supplementation differentially affected various bacterial populations. GTE, EGCG, and CAT attenuated HF-induced increases in *RuminiClostridium*, *Clostridium* cluster 1, *Blautia*, and *Lachnospiraceae* NK4A136 group. HF-induced decreases in *Akkermansia* were prevented by GTE and CAT, while increased levels of *Lactobacillus* and *Enterorhabdus* were observed in tea-supplemented groups. Specific changes in *Oscillibacter*, *Ruminococcaceae* UBA1819, and *Parasutterella* were noted in the HF+GTE and HF+CAT groups.<br>Subjects in the High fat diet added GTE or EGCG group had reduced blood glucose and insulin.
[138]	Female albino hairless mice (Skh:HR-1, 6–8 weeks old)	1% (w:w) green tea extracts (commercial) in diet each day	Female albino hairless mice were divided into three groups: Control (NOR), UVB exposed (UVB), and UVB-exposed with a diet containing 1% GTE (U+GTS). UVB Exposure Mice were exposed to UVB radiation three times a week, with increasing doses over time. NOR and UVB groups were fed a control diet, while the U+GTS group was fed a diet with 1% GTE. After 10 weeks, liver and large intestine samples were collected and stored for analysis. Intestinal Microbial Community Analysis included DNA extraction, PCR, and next-generation sequencing to analyze the intestinal microbiome. Extraction of large intestinal contents for metabolite profiling using UPLC-Q-TOF-MS and GC-TOF-MS.	UVB exposure slightly reduced microbial diversity, while green tea supplements increased diversity compared to other groups. UVB exposure impacted the microbiome and metabolome in the large intestine, and GTS supplementation influenced these effects.<br>Light reductions are shown in UVB-exposed groups compared to the control (NOR), while the UVB+GTS group showed enhanced diversity. At the phylum level, UVB exposure decreased *Bacteroidetes*, *Proteobacteria*, and *Verrucomicrobia* but increased *Firmicutes* relative to the NOR group. However, in the UVB+GTS group, all phyla, except *Firmicutes*, were higher compared to the other groups. UVB exposure increased *Lactobacillus* and *Lactococcus*, while GTS enriched *Allobaculum*, *Parvibacter*, *Lachnoclostridium*, and taxa from *Ruminococcaceae* and *Lachnospiraceae* families.<br>The correlation between specific bacteria and metabolites suggests potential interactions influencing the gut environment. Certain bacterial classes showed high correlations with specific metabolites, especially bile acids, affected by UVB and GTS.

	[139]	C57BL/6 female mice (20 ± 2 g, 7–8 weeks)	5 mg green tea water extracts powder /kg bodyweight each day	18 mice were divided into three groups, and received different substances through gavage for four weeks, then their feces were collected for bacterial analysis and used to create daily fecal material for transplanting into other mice. Thirty recipient mice were divided into Health, DSS, Normal_DSS, GTE_DSS, and DTE_DSS groups, given different treatments (a compound causing colitis or received fecal bacteria from the donor mice). After eight days of treatment, their colons were examined for length, tissue staining, and protein expressions related to inflammation and gut health. Fecal samples underwent DNA extraction and microbial analysis to understand changes in the gut microbiome.	The fecal microbiota from the GTE-treated donor mice significantly ameliorates colitis-related symptoms (colonic inflammation and gut microbiota dysbiosis).<br><br>GTE reduced the relative abundance of *Turicibacter* and *Romboutsia*, which showed high relative levels in patients or mice models of colitis and were associated with the progression of colitis.<br><br>GTE significantly reduced the relative abundance of *Allobaculum* (positively correlated with obesity).
	[140]	Germ-free C57BL/6J mice (6 weeks old)	0.1% (w/w) Green Tea Polyphenols<br><br>(water extracts) each day	10 kg of tea powder was brewed with 160 L of distilled water at 96°C for 40 minutes to prepare GTP. Germ-free mice were inoculated with fecal suspensions from eight healthy volunteers to establish a human flora-associated (HFA) mouse model. Mice were housed in a gnotobiotic isolator and divided into two groups: High-Fat Diet with water (HFD) and High-Fat Diet with GTP (HFD-GTP). After a 2-week adaptation to a high-fat diet, the HFD-GTP group received GTP (0.1%, w/w) for the first 3 weeks, followed by the high-fat diet without GTP. Body mass was recorded throughout the study, and fecal samples were collected at different time points (GTP-0 to GTP-4) for DNA extraction and 16S rRNA gene sequencing.	Treatment with GTP for 3 weeks resulted in a significant increase in *Bacteroidetes* and *Proteobacteria*, accompanied by a decrease in *Firmicutes* ($P < 0.05$). Even after discontinuation of GTP, certain communities, such as *Bacteroidetes* and *Proteobacteria*, continued to increase, while *Firmicutes* exhibited a decreasing trend during the 4th week.<br><br>GTP treatment significantly increased the relative abundance of *Ruminococcaceae* and decreased *Veillonellaceae*.<br><br>Certain genera, including *Megamonas*, *Roseburia*, *Butyricimonas*, and *Catenibacterium*, exhibited significant increases with GTP intervention. GTP intake may positively impact the stability of specific gut microbiota in an environment-triggered microbial imbalance.
**Oolong Tea**	[114]	Young adult (6-week-old) male C57BL/6J mice	0.1% (w/w) purified Oolong Tea Polyphenols (water extracts) each day	Young adult (6-week-old) male C57BL/6J mice were initially colonized with gut microbial communities derived from fresh fecal samples of 6 healthy volunteers. The mice were housed under a regular light/dark cycle for 7 days, then randomly divided into three groups: a regular light/dark cycle group (CONT), a constant dark group (CD), and a constant dark group with OTP treatment (CD-OTP). The 16S rDNA V3-V4 region of each sample was amplified using primers targeting the 16S rRNA gene region, and paired reads were merged using FLASH before sequencing. Total RNA was extracted from cecal contents and liver using Trizol, following the manufacturer's protocol. RNA quantity and purity were analyzed using a Bioanalyzer 2100 and RNA 1000 Nano LabChip Kit.	A significant decrease in *Firmicutes* of the CD-OTP mice and a relative increase of *Bacteroidetes* were observed compared to those of the CD group, and the *Firmicutes*/*Bacteroidetes* ratio also showed a significant decrease (86.47 vs 112.75%, respectively).<br><br>OTP can significantly improve the suppression of phylum-level cyclical changes caused by continuous darkness (ADJ.Ps are 9.28×10^{-7} for *Firmicutes*, 8.5×10^{-3} for *Bacteroidetes*, 0.01 for *Proteobacteria*, and 0.20 for *Actinobacteria*).

[115]	young adult (6-week-old) male C57BL/6 J mice	2% (w/v) purified Oolong tea polyphenols (water extracts) each day	Male C57BL/6 J mice (6 weeks old) were colonized with a microbial community from freshly voided fecal samples of six healthy human volunteers (3 females, 3 males, 25–27 years old). The samples were placed in an anaerobic environment, and aliquots were gavaged into each mouse. The mice were kept at 22-24 °C with a 12-hour light-dark cycle and 50 ± 10% humidity. After 7 days of adaptation on a high-fat diet, they were randomly divided into three groups: low-fat diet, high-fat diet (HFD) with water, and high-fat diet with oolong tea (HFD-OT). Fecal samples were collected at 0, 2, 4, and 8 weeks. DNA was eluted in 50 μL of elution buffer and stored at −80 °C for PCR assays. The 16S rDNA V3-V4 region was amplified for sequencing.	The oolong tea treatment significantly increased the relative abundance of *Bacteroidetes*, while there was a significant decrease in *Firmicutes*, additionally, the ratio of *Firmicutes/Bacteroidetes* was dramatically decreased.<br>The relative abundance of *Prevotella*, *Bacteroides* and *Megamonas* were increased by oolong tea intervention, accompanied by a reduction in *Lachnospira, Eubacterium, Clostridium, Ruminococcus* and *Phascolarctobacterium.*
[121]	The 8-week-old C57BL/6J male mice	200 mg/kg body weight Black tea powder (water extracts, concentrated and dried) each day	All experimental mice were fed the Lieber-DeCarli control liquid diet ad libitum for 5 days. Mice were then randomly assigned to ethanol-fed and control groups based on body weight. The ethanol-fed group received the Lieber-DeCarli ethanol diet, while the control group continued on the control diet. After 6 days, the ethanol-fed group was further divided into a model group and six tea extract supplement groups. The tea extract groups were given the ethanol diet with 4% ethanol and different tea extracts (10g tea in 100 mL water, concentrated and dried) for 4 weeks. The model and control groups received distilled water by gavage. Fecal samples were collected and stored at −80°C. After 9 hours of fasting, all mice were weighed, anesthetized, and sacrificed to collect blood and liver samples amplification and sequencing of the 16S rRNA genes were conducted.	After intervention with Oolong tea extract (OTE), the increase in intestinal flora richness and decrease in diversity caused by alcohol exposure were significantly restored.<br>Treatment with oolong tea (Tieguanyin) extract significantly inhibited alcohol-induced reduction of *Verrucomicrobia* and *Actinomycetes*<br>the treatments of olong tea, extracts significantly decreased the relative abundance of *Bacteroidetes*, and significantly increased that of *Firmicutes* and the ratio of *Firmicutes/Bacteroidete*
[112]	Sterile male mice C57BL/6J	0.1% (w/w) Oolong tea polyphenols (water extracts) each day	Mice were individually housed in a sterile environment at 22.3°C with 55.5% humidity, under a 12-hour light-dark cycle. After 7 days of acclimatization, 18 mice were randomly assigned to four groups: CT, CD, VF, and OTP. The CT group remained under normal light-dark conditions, while the CD and VF groups were kept in complete darkness. The OTP group was also kept in darkness but received daily OTP treatment. The CT, CD, and VF groups were given sterile water daily. Weekly measurements of body weight, water, and food intake were recorded. DNA was extracted using the E.Z.N.A.® Stool DNA Kit, and total DNA was analyzed by PCR.	OTP improved the structural disorder of the intestinal flora caused by continuous darkness, thereby modulating the production of metabolites related to pyruvate metabolism, glycolysis/gluconeogenesis, and tryptophan metabolism to alleviate the steady-state imbalance.<br>OTP treatment could significantly improve the decrease in the relative abundance of *Prevotella* and *Bacteroides*<br>OTP significantly improves intestinal microbial diversity in circadian rhythm disorder mice model

[110]	Six-week-old male C57BL/6J mice (6–8 weeks old, 23 ± 2 g)	200 mg/kg body weight oolong tea polyphenols (purified) each day	Mice were housed in groups in a sterile barrier facility at 20–24 °C with 60 ± 5% humidity and fed autoclaved normal chow. After 1 week of acclimatization under a 12/12 h light–dark cycle, they were randomly divided into 3 groups (15 mice each): control (CT) with a 12/12 light–dark cycle, continuous dark (CD) in constant darkness, and OTP group given OTP for 4 weeks in darkness. The CT and CD groups received 0.2 mL of sterile saline by gavage. Body weight, food, and water intake were recorded weekly. Fecal samples were collected at 0 and 4 weeks and stored at −80 °C for 16S rDNA gene sequencing and untargeted metabolomic analysis of fecal and cecum contents.	OTP treatment restored the gut microbiota disruption including up-regulated the relative abundance of *Akkermansia* and *Muribaculum*, and down-regulated Desulfovibrio. OTP group had more Observed species, Chao1, Shannon and Simpson index than the CD group.<br><br>OTP reversed the microbial differences within the groups due to diurnal disturbances.<br>The greater inhibitory effect of OTP on the growth of the *Firmicutes* and the positive regulation of the gut microbiota.
[116]	6-week-old male C57BL/6 J mice	0.1% (w/w) oolong tea ground powder and purified EGCG3″Me each day	Young adult mice were colonized with microbial communities from fresh fecal samples of five healthy volunteers. Samples were kept in an anaerobic environment, and a portion was introduced into the mice via gavage within one hour. After a 7-day high-fat diet adaptation, mice were divided into three groups: low-fat diet (LFD), high-fat diet (HFD), and high-fat diet with EGCG3″Me (HFD-EGCG3″Me). Fecal samples from the HFD-EGCG3″Me group were collected at 0, 2, 4, and 8 weeks. DNA was extracted, and PCR amplification targeting the 16S rRNA gene was performed in a 50 µL reaction mixture. Raw DNA fragments were merged using FLASH (V1.2.7).	Feeding EGCG3″Me can significantly reduce fecal microbiota diversity<br>After EGCG3″Me treatment, the relative abundance of *Bacteroidetes* and *Proteobacteria* significantly increased, while the relative abundance of *Firmicutes* decreased significantly; the ratio of *Firmicutes* to *Bacteroidetes* significantly decreased.<br>*Bacteroides*, *Sutterella, Megasphaera, Faecalibacterium, Mitsuokella, Coprococcus and Roseburia* were increased by the EGCG3″Me intervention, accompanied by a reduction in the relative abundance of the *Prevotella, Lachnospira, Haemophilus, Oscillospira, Turicibacter, Odoribacter* and *Bilophila.*
[118]	6-week-old male C57BL/6J mice	0.1% w/w purified oolong tea polyphenols (water extracts) each day	Mice were initially colonized using microbial communities present in fecal samples from six healthy volunteers. Processed fecal samples were introduced into each germ-free mice by gavage. The mice were adapted to the environment on a high-fat diet for 7 days, and then randomly divided into 5 groups: low-fat diet group (LFD), high-fat diet group (HFD), high-fat diet plus OTP group (HFD-OTP), and high-fat diet plus OTC. group (HFD-OTC), high-fat diet plus phospholipid group (HFD-PP). Fecal samples were collected 1, 2, 4, and 8 weeks after being divided into each group. Bacterial cells were counted using Fluorescent in situ hybridization (FISH) technique.	Populations of *Lactobacillus*/*Enterococcus spp*. Increases were seen in all HFD treatment groups.<br>OTP showed a significant proliferative effect on *Lactobacillus*/ *Enterococcus spp* starting in the second week<br>Compared with the HFD group, OTP treatment showed a significant reduction in the levels of *Bacteroides prausnitzii* and *Clostridium histolytica* starting from the second week.

[117]	6-week-old male C57BL/6J mice	0.1% (w/w) oolong tea ground powder and purified EGCG3″Me each day	Germ-free mice were colonized with microbial communities from fecal samples of six healthy volunteers. Processed fecal samples were introduced via gavage. After a 7-day adaptation on a high-fat diet (HFD), mice were randomly divided into three groups: low-fat diet (LFD), HFD, and HFD with added EGCG3″Me (HFD-EGCG3″Me). Body weight was recorded after feeding the different diets. Fecal samples from the HFD-EGCG3″Me group were collected at 0, 2, 4, and 8 weeks. DNA was extracted, amplified using primers targeting the 16S rRNA gene, and sequenced. Read pairs were combined with FLASH (v1.2.8) for gut microbiota analysis.	After 8 weeks, EGCG3″Me treatment significantly decreased the relative abundance of *Firmicutes* and significantly increased the relative abundance of *Bacteroidetes*. In addition, the ratio of *Firmicutes* to *Bacteroidetes* significantly decreased. *Prevotella*ceae and *Bacteroidetes* showed an increasing trend during EGCG3″Me treatment and reached a maximum value after 8 weeks. The relative abundance of *Prevotella*ceae was significantly increased after feeding EGCG3″Me. Increase. In contrast, after feeding mice EGCG3"Me for 8 weeks, the abundance of *Ruminococcaceae*, *Lachnospiraceae*, and Veillonellaceae was lower. Belonging to the phylum *Firmicutes* The abundance of
[119]	Twenty-four 8-week-old cleaning Wistar male rats	500mg/kg oolong tea water extract added to drinking water each day	All rats were housed with free access to food and water in a controlled environment (25 ± 1°C, 70–75% humidity, 12-hour light-dark cycle). After acclimatization for 1 week, the NC group received Shoobree common standard feed for 9 weeks on a regular diet; MC, GT (feed green tea), and OLT (feed oolong tea) groups received with high-salt chow for 9 weeks to induce hypertension. The GT and OLT groups also received green tea and oolong tea extracts in their drinking water, respectively, while the NC and MC groups received distilled water. Body weight and systolic blood pressure were measured at the start and end of the treatment.	the intervention of GT and OLT alleviated the decline in the OTU richness and α-diversity caused by the long-term high-salt diet<br>At the phylum level, OLT supplementation significantly decreased *Firmicutes* and significantly increased *Bacteroidetes*. Accordingly, OLT supplementation could substantially reduce the ratio of *Firmicutes* to *Bacteroides*.<br>At genus level, the OLT group was characterized by specific and significant enrichment for the *Allobaculum, ParaPrevotella, Oscillospira, Bifidobacterium,* and *Ruminococcus genera*.<br>OLT considerably increased the relative abundance of *Allobaculum* and *Bifidobacterium*.
[120]	Seventy-two male specific-pathogen-free grade Kunming mice, with a weight of 35–40 g at 6 weeks of age	800 mg/kg·d oolong tea water extract, administered by gavage once a day for two consecutive weeks.	After a 1-week acclimation, mice were randomly divided into nine groups of eight: (1) control (0.5% CMC-Na); (2) model (potassium oxonate and adenosine); (3) allopurinol; (4)-(9) tea groups, with TWE treatment equivalent to an adult consuming 12–15 g of tea daily. The experiment lasted 5 weeks. All groups except the control received potassium oxonate and adenosine daily. The allopurinol group and the six tea groups were gavaged daily for two consecutive weeks. After 5 weeks, mice were fasted for 12 hours, anesthetized, and whole blood was collected via cardiac puncture.	While the relative abundance of *Bacteroidota* significantly decreased after TWE treatment, the relative abundance of the *Firmicutes/Bacteroidota* ratio significantly decreased after Tea treatment.<br>TEW treatment reduced the relative abundance of *Bacteroidetes* and the relative abundance of *E. coli.*

Black Tea	[126]	ICR mice, aged 8 weeks and weighing 22 ± 2 g (32M and 32F).	25 mg/ml black tea water brew each day	To prepare black tea brew, add 5 g of black tea to 50 mL of boiling water. All animals were kept in clean, sterile cages under controlled conditions (12-hour light/dark cycle, 60% humidity, 25°C). After a 1-week chow diet adaptation, they were divided into normal, HFD, HFD + BT, and HFD + green tea groups. Body weight was recorded daily. After 16 weeks, mice were fasted overnight, anesthetized with sodium pentobarbital, and sacrificed by cervical dislocation to obtain blood, organs, and tissues. Blood samples were centrifuged at 8000 rpm for 15 minutes at 4°C to isolate serum, while cecal and colon contents were collected for 16S rRNA gene sequencing and metabolomics analysis.	Slightly reduced alpha diversity of intestinal microbiota, slightly improved insulin resistance and glucose and lipid metabolism<br>The gutbacteria, *Clostridium_XlVa* and *Lawsonibacter,* enriched by black tea displayed no correlation with related metabolites and index.<br>The improvement of metabolic disorder by black tea is independent of its regulation on gut microbiota
	[121]	The 8-week-old C57BL/6J male mice	200 mg/kg b.w. black tea extracts powder (dried water extracts) each day	Mice were initially fed a Lieber-DeCarli control liquid diet for 5 days. They were then divided into ethanol-fed and control groups based on body weight. Ethanol-fed mice received Lieber-DeCarli ethanol liquid diet (4% ethanol), while control mice received the control diet. After 6 days, ethanol-fed mice were further divided into a model group and six tea extract groups, which were given various tea extracts for 4 weeks, while the model and control groups were given distilled water. After 4 weeks, fecal samples were collected and stored at −80 °C. Mice were then fasted for 9 hours, weighed, anesthetized, and sacrificed to collect blood and liver samples for further analysis.	Black tea extract (BTE) treatment had minimal effects on gut microbiota richness and diversity.<br>Treatment with black tea did not prevent changes in the gut microbiota.<br>Black tea significantly reduced the relative abundance of *E. coli.*<br>The relative abundance of *Verrucomicrobia, Para Bacteroidetes* and *Actinobacteria* was increased in black tea.
	[19]	he 8-week-old C57BL/6J male mice	200 mg/kg b.w. black tea extracts powder (dried water extracts)	Mice were housed in specific-pathogen-free conditions with controlled light, temperature, and humidity, and had ad libitum access to diet and water. After a one-week acclimatization, 8-week-old mice were randomly divided into 8 groups based on body weight. The first group was fed a normal diet (ND group), while the second group received a high-fat diet (HFD group). The remaining groups were given HFD supplemented with various tea extracts at 200 mg/kg b.w./d for 15 weeks via intragastric gavage, with the ND and HFD groups receiving deionized water (10 mL/kg b.w./d). Body weight and food intake were measured weekly. For 16S rRNA analysis, PCR amplification, product measurement, mixing, recovery, and DNA fragment construction were performed.	Black tea interventions could significantly inhibit the elevation of *Firmicutes* to *Bacteroidetes* ratio<br>Selenium-enriched black tea and black tea supplementation could markedly attenuate the increase in the relative abundance of *Firmicutes*.<br>Compared with the HFD group, selenium-enriched black tea treatments could significantly reduce the relative abundance of *Actinobacteria*.<br>The relative abundance of *Allobaculum* and *Lactobacillusin* the HFD group showed an increasing trend, but that the intervention of black tea significantly inhibited the elevated trend.<br>The elevation in the relative abundance of *Roseburia* induced by HFD could be markedly inhibited by supplementation of BT.

[125]	6-week C57BL/6J male mice	760 mg/Kg. Average energy intake<br><br>Purified black tea water extracts	Mice were kept on a 12-hour light/dark cycle with free access to food and water in a pathogen-free environment. The treatment group received daily intragastric administration. Six-week-old male donor mice on a high-fat diet were given black tea infusion orally for 4 weeks. Daily, 100 mg of their feces was collected, mixed with 1 mL sterile saline, and centrifuged at 3000 × g for 5 minutes. The supernatant was used immediately for transplantation to maintain bacterial composition. Six-week-old HFD-fed C57BL/6J male mice received 0.1 mL of the fecal inoculum daily via oral gavage for 18 weeks. 16S rRNA gene sequencing was performed.	Similar changes occurred in the relative abundance of different taxonomic levels, with the Clostridiaceae_1 family and the genera *Turicibacter*, Vagococcus and Eubacterium prostanol being enriched in the black tea intervention group.<br><br>black tea in HFD-fed mice differentially reversed the relative abundances of 56 OTUs altered by HFD feeding, of which 18 were enriched (Eubacterium coprostanoligenes, *Lactobacillus* gasseri, *Bacteroidales* S24-7, etc.) and 38 were reduced (*Roseburia* spp., *E. coli*, *Clostridium* sp. ND2, *Lactobacillus* casei, *Eisenbergiella*, *Ruminococcaceae* UCG-009, *Desulfovibrio*, *Akkermansia*, *Lachnospiraceae* UCG-006, *Blautia*, etc.).
[127]	48 male C57BL/6J mice at 6–7 weeks of age (body weight: 16–18 g)	320 mg/kg body weight ethanol-water black tea extracts each day	Black tea extracts were prepared by extracting 500g of tea leaves with 2L of 75% ethanol-water solution, evaporating the ethanol under vacuum, suspending the residue in 500mL water, and extracting with chloroform to remove caffeine. The extract was then eluted with methanol and dried using a rotary evaporator. Mice were divided into four groups, fed a low-fat/high-sucrose diet with mixed tea extract, and monitored for body weight and food consumption over 4 weeks. After euthanasia, tissues were collected, weighed, and stored at −80°C for analysis. DNA was extracted from the tissues and amplified using barcoded forward primers targeting the V4 region of the 16S rRNA gene, with amplification confirmed by agarose gel electrophoresis.	BTP increased the relative proportion of *Pseudobutyrivibrio* and intestinal formation of short-chain fatty acids (SCFA).<br>There is significant increase in the relative proportion of cecum phyla *Bacteroidetes* and decrease in *Firmicutes* and Actinobacter in mice fed the BTP diet.<br><br>On the genus level feeding, the BTP diets were associated with a significant increase in relative proportion of *ParaBacteroides*, *Bacteroides*, and *Prevotella* and a significant decrease in several genera from the phylum *Firmicutes* such as *Roseburia*, *Lactobacillus*, *Blautia*, Anaerostipes, Shuttleworthia, *Bryantella*, Lactococcus, and Acetitomaculum as well as a significant decrease in *Collinsella* from the *Actinobacter phylum.*
[124]	48 C57BL/6 mice (male, 6 weeks)	2.0% black tea ground powder [w/w] each day	Mice were housed in a specific pathogen-free environment with controlled conditions and received one of four diets after 1 week of acclimation: LFD, HFD, HFD with 2% KBT powder, or HFD with 2% DBT powder. Body weight was recorded weekly over a 15-week period, and food and water consumption were monitored every other day. Feces were collected bi-weekly and stored at -80°C. After 15 weeks, the mice were sacrificed under anesthesia. Fecal genomic DNA was extracted, with the hypervariable V3-V4 region of 16S rRNA selected for amplification. Alpha diversity analysis assessed species complexity, and beta diversity analysis was used to evaluate species diversity in the samples.	A HFD causes dramatic changes in the gut microbiota. Compared with LFD animals, HFD mice had an increased proportion of *Firmicutes*, a decreased proportion of *Bacteroidetes*, and a significant increase in the proportions of *Firmicutes* and *Bacteroidetes*. However, black tea treatment did not significantly affect this ratio.

[123]	Male SD rats (6 weeks-of-age, n = 14)	1.5 g/kg body weight black tea powder (suspended in distilled water) per day	After a 1-week acclimatization period, rats were randomly assigned to two groups: a control group (N_C) and a black tea group (N_B). The N_B group received black tea powder (1.5 g/kg body weight per day) via intragastric gavage for 4 weeks, while the N_C group was administered the same volume of distilled water. Weekly recordings of food intake and body weight were made. Prior to sacrifice, rats were fasted for 12 hours with access to water. Intestinal contents were collected from the lower cecum, stored at -80°C, and bacterial genomic DNA was extracted. The V3-V4 hypervariable region of the 16S rRNA gene was amplified by PCR, and PCR products were purified and quantified using a Qubit 3.0 instrument. Illumina MiSeq sequencing was performed after quality control analysis.	Black tea significantly increased the alpha diversity of the gut microbiota.<br><br>black tea did not significantly affect the relative abundance of *Proteobacteria*. black tea significantly increased the relative abundance of *Firmicutes* and decreased the relative abundance of *Bacteroidetes*, resulting in an increased *Firmicutes*/*Bacteroidetes* ratio.<br><br>black tea resulted in a decrease in the relative abundance of the *Lactobacillus* genus, which is considered a probiotic.
[120]	Seventy-two male specific-pathogen-free grade Kunming mice, with a weight of 35–40 g at 6 weeks of age	800mg/kg·d black tea water extracts (freeze-dried powder)	The study involved preparing Boil Tea Extract (BTE) by boiling black tea in water at a 1:20 ratio for 30 minutes, repeating this process three times, then filtering, freezing, and freeze-drying the liquid. Mice were acclimated for one week and then randomly divided into nine groups of eight. Groups included a control (0.5% CMC-Na), a model (potassium oxonate and adenosine), an allopurinol group, and six tea groups. The tea treatment dose was equivalent to 12–15 g of tea per day for humans. After 5 weeks of daily gavage (except the control group), mice were fasted for 12 hours, anesthetized, and blood was collected via cardiac puncture.	While the relative abundance of *Bacteroidota* significantly decreased after TWE treatment, the relative abundance of the *Firmicutes*/*Bacteroidota* ratio significantly decreased after TWE treatment.<br><br>TEW treatment reduced the relative abundance of *Bacteroidetes* and the relative abundance of *E. coli*.
[122]	Four-week-old, male Wistar rats (n = 21)	10 g/kg Black tea extracts (ethanol aqueous freeze-dried powder) each day	Black tea extract (BTE) was prepared by soaking black tea in a 395 g/L ethanol aqueous solution, filtering, evaporating under reduced pressure, and freeze-drying. The dry sample was dissolved in water, extracted with chloroform, and freeze-dried to obtain decaffeinated BTE. Rats were acclimated for 1 week on commercial chow, then divided into three groups and fed control, green tea extract (GTE), or BTE diets with free access to water for 3 weeks. Feces were collected weekly, and on the final day, rats were euthanized with carbon dioxide. The cecum was excised, stored at −40°C, and DNA was extracted. Quantitative real-time PCR was used to quantify 16S rRNA gene copies for total eubacteria.	The BTE diet showed significant reductions in *Clostridium* subcluster XIVa and *Clostridium* cluster XI, the relative abundance of *Clostridium* subcluster XIVa was decreased.<br><br>The BTE diet slightly raised median values of the *Bifidobacterium* abundance.

	[128]	Eight-week-old male ICR mice weighing 35–40 g	50 mg kg/1 Black tea theaflavins (commercial) every day	Mice were divided into five groups: Control (CK), Aging d-galactose-treated (Model), Vitamin C-treated (VC), and TFs-treated (TF). After a 12-hour fast, mice were anesthetized with 5–7% chloral hydrate and sacrificed by cervical dislocation. Blood, brain, intestine, and feces were collected for analysis. DNA was extracted using the E.Z.N.A. Stool DNA Kit, and PCR amplification was performed on bacterial 16S rRNA, followed by purification and quantification.	TF treatment led to the relative abundances of *Actinobacteria* and the ratio of *Firmicutes* to *Bacteroidetes* increased, and the relative abundances of *Bacteroidetes* and *Proteobacteria* decreased.<br><br>At the genus level, d-galactose reduced the relative abundances of *Bifidobacterium* and *Clostridium* and the increased relative abundances of *Bacteroides*, *Bacteroidetes*, *Roseburia*, and *Lachnospiraceae*. The relative abundances of these genera were effectively recovered by TFs.<br><br>Chao1, Otus, and Shannon significantly decreased ($p < 0.01$) and Simpson significantly increased ($p < 0.01$) in the fecal samples from the d-galactose-induced aging mice, indicating a lower microbiota community diversity induced by d-galactose. The intake of TFs restored the gut microbiota α-diversity.
**Pu-erh tea**	[133]	3-week-old C57BL/6J male mice	3 mg/mL instant Pu-erh tea (PT) water infusion (450 mg/Kg/day)	Different groups were established based on diet composition and tea interventions: Control group (received normal chow diet and water or Pu-erh tea infusion), High-fat diet group, HFD with PT infusion group, etc. Various doses of PT infusion, theabrownin, bile acids, and other compounds were administered per kilogram of body weight per day. Interventions were conducted for different durations ranging from 4 weeks to 46 weeks. Regular measurements of body weight, food intake, tea/water consumptions, blood samples, and tissue collections were made throughout the experiments. Used fecal transplantation from HFD-fed mice or HFD + Theabrownin-treated mice to germ-free mice.	OTUs in the *Lactobacillus*, *Bacillus*, *Enterococcus*, *Lactococcus*, *Streptococcus*, and *Leuconostoc* genera were reduced, showing the same differential tendencies in both the ND + PT and HFD + PT groups of mice.<br><br>PT altered the composition of gut microbiota in mice, reducing the populations of specific bacteria known for producing enzymes involved in bile acid metabolism, such as BSH.

[131]	8-week-old male C57BL/6N mice	0.4% and 1% (w/v) Ripened Pu-erh tea water extracts each day	100g of Pu-erh tea was extracted with 1000mL boiling water for 5 minutes, lyophilized, and stored at 4°C. Mice were divided into groups: standard chow, standard chow with varying PTE concentrations, HFD, and HFD with varying PTE concentrations. Each group (10 mice) received their respective treatments for 8 weeks. Parameters assessed included body weight, liver and fat pads, oral glucose tolerance using an ACCU CHEK glucometer, plasma LPS levels via ELISA, and lipid profiles (HDL-C, LDL-C, TC, TG) using commercial kits. Fecal microbial DNA was extracted, and 16S rRNA variable 4 region was sequenced for alpha and beta diversity analysis. Data were expressed as mean ± standard deviation, with statistical significance determined by one-way ANOVA and Tukey test using SPSS 19.	PTE-supplemented mice gained significantly less weight compared to HFD-fed mice. PTE supplementation reduced the abundance of *Mucispirillum schaedleri* in HFD-fed mice but increased the abundance of *Bacteroides Acidifaciens*, *ParaBacteroides goldsteinii* and *ParaBacteroides distasonis*. In addition, subjects from different PTE diet groups showed significantly different microbiota. And *Lactococcus lactis* showed a significant positive correlation with obesity-related indicators.
[132]	Male Wistar rats fed a high-fat and high-sugar diet (HFSD) to induce obesity	0.15-g/kg and 0.4-g/kg body weight PTE (aqueous extracts) each day	Male Wistar rats were fed a high-fat and high-sugar diet to induce obesity and divided into five groups: one control group receiving only the diet and four groups receiving different doses of aqueous extracts from fermented and non-fermented PET. The extracts were administered at doses of 0.15 g/kg and 0.4 g/kg body weight. After 5 weeks, blood samples were collected for serum analysis, and proteins from cecal samples were analyzed via mass spectrometry to assess microbiota changes.	PETex increased the biodiversity of cecal bacterial communities in rats fed HFSD. Administration of raw and ripe PETex resulted in increased relative abundances (16SRA) of *Firmicutes*, particularly the genus *Lactobacillus*, and *Actinobacteria*, including the genus *Bifidobacterium*.<br><br>Both 16SRA and PRA for *Bacteroidetes*, including the genus *Bacteroides*, decreased after raw and ripe PETex administration.<br><br>PETex affected core functional genes and enzymes in the cecal microbiota, impacting carbon metabolism and genetic information processing.<br><br>Enriched enzymes from *Akkermansia muciniphila* in response to PETex administration were identified.
[141]	Male Sprague-Dawley rats (weighing 180–220 g)	Pu-erh tea water extracts 0.16 g/mL, 15 mL/kg/day	Pu-erh tea samples were cut, soaked in boiled distilled water three times, and extracted. Male Sprague-Dawley rats (40 total) were divided into four groups: Control, GTE (0.2 g/mL), PTE (0.16 g/mL), and GCPE (0.04 mL). Each group was gavaged twice daily for 28 days. Fecal samples were collected for gut microbiota analysis, involving DNA extraction, 16S rRNA gene sequencing, and data analysis including alpha and beta diversity, LEfSe analysis, and identification of enriched genera.	Long-term consumption of PTE did not significantly affect overall gut microbiota in healthy rats.<br><br>Some bacteria that are common in the fermentation process of Pu-erh tea were also found to be enriched in the gut microbiota of rats in the PTE group, including *Bacillus*, *Brachybacterium*, *Paenalcaligenes*, and *Jeotgalicoccus*.<br><br>Ganpu tea showed a capacity to stimulate probiotics in the gut microbiota.

[129]	42 male C57BL/6 mice (6 weeks of age, body eight 20.4 ± 1.0 g)	600 mg/kg/d and 300 mg/kg/d raw or ripened Pu-erh tea water extracts	Male mice were divided into six groups and given various diets, including a normal-chow diet (ND), a high-fat diet (HFD), and HFD with different doses of raw or ripened Pu-erh tea extracts via intragastric gavage. After feeding, parameters such as body weight, fat accumulation, liver weight, food intake, glucose tolerance, serum lipid profile, oxidative stress markers, inflammatory cytokines, gene and protein expression related to lipid metabolism, and gut microbiota were measured.	High fat diet disrupted gut microbiota composition, reducing richness and diversity.<br>While PETe restored the diversity and shifted the microbiota composition towards that of the normal-chow diet group, especially at higher doses.<br>PETe reversed the altered abundance of specific bacterial groups induced by HFD, such as increasing beneficial bacteria like *Bacteroides* and *Akkermansia* while reducing harmful ones like *Lactobacillus* and *Streptococcus*.
[135]	Six-week-old C57BL/6J male mice	Daily doses of 750 mg/kg of body weight Pu-erh tea (aqueous extraction) each day	Six-week-old male C57BL/6J mice, housed in groups of three or four in a controlled environment with ad libitum access to food and water, were divided into nine groups based on body weight and fasting glucose levels. Control groups received water (NCD or HFD), while seven treatment groups received various compound doses. Assessments included levels of TCHO, TG, HDL-C, LDL-C, liver TG, LPS, and insulin. Metagenomic and microbial DNA were analyzed via PCR-based techniques, gene expression via reverse transcription-qPCR, and protein expression using specific antibodies.	PE and TP significantly reduced fasting blood glucose and reduced mean fat cell size.<br>PE and TP treatment significantly reduced HFD-induced intestinal inflammatory response, including infiltration of inflammatory cells and mRNA expression of inflammatory factors.<br>PE, TP restored the structure of HFD-induced intestinal microbial community.PE and TP treatments increased the rectal *Fusobacterium*/*Clostridium globosum* group, *F. prausnitzii*, *A. muciniphila*, *Bifidobacterium spp.*
[134]	Female C57BL/6 mice (8 weeks old; 17–20 g)	3%, 6% and 9% (w:w) Ripened Pu-erh tea water extracts each day	Female C57BL/6 mice (8 weeks old) were divided into six groups: Control, DSS, and three RPT dosage groups (low, moderate, and high). RPT was administered for 7 days before DSS treatment, which lasted 1 week. Tissues, serum, and cecal contents were collected for analysis. Donor mice were gavaged with Pu-erh tea or water, and their fecal pellets were used for fecal microbiota transplantation (FMT) into recipient mice pre-treated with antibiotics. After 1 week, recipients were treated with DSS for 1 week, and tissues were collected. RNA was extracted from colon tissue for qPCR analysis, and genomic DNA from cecal contents was used for 16S rRNA gene sequencing, taxonomy assignment, diversity analysis, and NMDS of ASVs.	Pu-erh tea rescued the intestinal dysbiosis induced by dextran sulfate sodium treatment. Pu-erh tea treatment reversed the situation that DSS-induced increase in the relative abundance of *Aspergillus phylum* and the decrease in the thick-walled phylum in mice with colitis.<br>At the genus level, Pu-erh tea treatment significantly increased *Enterobacteriaceae* and *Helicobacteraceae*.
[142]	24 mice (12 males and 12 females)	0.15 ml Pu-erh tea water extract each day	Pu-erh tea was prepared by boiling 3g of tea in 150mL distilled water for 30 minutes, with additional water added as needed, followed by filtration and centrifugation to obtain the aqueous extract. Twenty-four mice were housed under controlled conditions and divided into three groups: blank control (water), Pu-erh tea, and Dian Hong black tea. Mice were gavaged daily with their respective tea extracts or water for 15 days. Genomic DNA was extracted from cecal contents using the CTAB/SDS method, followed by PCR amplification of the 16S rRNA gene V4 region with unique barcodes for each sample, and sequencing of the amplified products.	Significant differences in microbial community structures among water, Pu-erh tea, and Dian Hong black tea-treated mice. Dominance of *Firmicutes* and *Bacteroidetes*, differences observed in the relative abundance of these phyla. At the genus level, *Lactobacillus*, *Lachnospiraceae*, *Ruminococcaceae*, *Alistipes*, *AlloPrevotella*, *Turicibacter*, *Bacteroides*, *Desulfovibrio*, *Faecalibaculum*, and *Parasutterella* were dominant.

	[130]	Fifty C57BL/6J male mice	400 mg/kg raw Pu-erh tea theabrownin (R-TB) or ripened Pu-erh tea theabrownin (F-TB) (purified) each day	Fifty C57BL/6J male mice were divided into five groups (ND, HFD, AL, R-TB, and F-TB) and fed their respective diets for 24 weeks. Parameters such as food intake and body weight were monitored. At the study's end, blood samples, liver and adipose tissues, and cecum contents were collected. Glucose tolerance was assessed through blood glucose measurements, while gut microbiota composition was analyzed via DNA sequencing. Quantitative RT-PCR was used for gene expression analysis in tissues.	Both R-TB and F-TB improved gut microbiota diversity and richness in obese mice induced by an HFD. These compounds restored the gut microbiota composition and structure altered by the high-fat diet. F-TB showed a greater potential in adjusting dysbiosis and normalizing *Firmicutes*/*Bacteroidetes* ratio compared to R-TB.<br><br>Both R-TB and F-TB influenced the metabolic profiles of gut microbiota, up-regulating glycan biosynthesis and cell motility, and down-regulating amino acid metabolism and energy metabolism.<br><br>F-TB was more similar to the normal diet group in terms of gut microbiota metabolic profiles compared to R-TB.
	[143]	male C57BL/6 J mice	0.25% (w/v) Pu-erh tea (water extracts freeze-dried powder) each day	The experiment used male C57BL/6J mice divided into water and RPT groups, which were further split into control with water (CK-W), control with RPT (CK-T), CRD with water (CR-W), and CRD with RPT (CR-T). Mice were fed Pu-erh tea for 40 days under different light conditions to simulate CRD. Various assays measured the effects of CRD and RPT on body weight, blood glucose, insulin levels, lipid profiles, liver enzymes, inflammatory cytokines, and analyzed liver and intestinal tissues, as well as gut microbiota.	Pu-erh tea significantly reduced CRD-induced increases in relative abundance in the *Lachnospiraceae NK4A136* group, the *Lactobacillus* group and the *Marvinbryantia* group.<br><br>At the genus level, Pu-erh tea restored abundance levels of *norank_f_ Muribaculaceae* and *Lactobacillus*, whereas CRD reduced these abundance levels.
	[144]	Pathogen-free C57BL/6 mice (8 weeks of age, weighing 20 ± 2 g)	0.1 or 0.4% (1 or 4 g/L, w/v) Pu-erh tea freeze-dried water extracts each day	Pu-erh tea was extracted using distilled water, freeze-dried, and stored for further use. Male C57BL/6 mice were randomly divided into four groups: health, alcohol, low-dose PTE (LPTE), and high-dose PTE (HPTE). Mice were given water or an ethanol solution daily by gavage. LPTE and HPTE groups received different concentrations of PTE in their drinking water. Fecal samples were analyzed for microbiomic and metabolomic changes. Liver tissues were examined for biochemical and histological indicators related to alcohol-induced damage.	Alcohol altered gut microbiota diversity and composition. PTE treatment reversed these changes, including the restoration of microbiota diversity and alterations in specific bacterial genera.<br><br>The *Firmicutes/Bacteroidete* ratio was significantly reduced by alcohol, and HPTE significantly reversed this trend. The alcohol significantly decreased the relative abundance of *norank_f_Muribaculaceae* and increased the relative abundance of *Bifidobacterium* and *Allobaculum*, whereas this situation was significantly reversed by PTE treatment.

Supplementary Table S2: Human fecal fermentation studies.

Tea Type	Reference	Subjects	Treatment	Methodology	Major findings
Green Tea	[145]	Fresh fecal samples donated by three adults with metabolic syndrome (two males and one female)	2% (w: v) GTE freeze-dried powder each day	The experiment comprised a 14-day equilibrium period, a 7-day pre-treatment phase, a 14-day treatment phase, and a 7-day washout phase (with GTE only during treatment). The temperature was kept at 37°C in an anaerobic environment. Daily HCM samples from each colon compartment were collected for metagenomics and metabolomics analysis. DNA from the samples was sequenced, and metabolites, free fatty acids, and phenolic compounds were extracted and analyzed using LC/MS.	There was an overall shift in gut microbiota composition at genus level after GTE treatment. *Escherichia*, *Klebsiella* surged in transverse, descending colon; *Bacteroides*, Citrobacter, *Clostridium* declined in all sections. In ascending colon, *Bilophila*, *Sutterella* decreased; *Enterococcus* abundance declined after GTE supplementation.<br><br>GTE supplementation significantly increased the production of nine free fatty acids by gut microbes in the HCM system.
	[153]	Human Colon Microbiota (from human fecal samples of two healthy volunteers)	0.67 mg/mL GTE freeze-dried powder (boil water extracts) each day	Crude dry green tea leaves were infused in boiling deionized water (1:20 w/w), filtered, and freeze-dried. A 0.67 mg/mL green tea extract was added to Gifu Anaerobic Agar plates and fermented using the HCM system. This was followed by an offline stomach process with pH adjustments and enzymatic digestion before adding the medium. The Human Colon Microbiota system simulated meal intake with fresh medium injections three times daily, allowing a 1-hour delay between vessel transitions for microbial metabolism. Metagenomic analysis of culture samples was performed via 16S rRNA gene sequencing, and metabolite extraction and targeted metabolic profiling, along with phenolic compound detection, were conducted.	The exposure of GTP to gut microbiota resulted in a significant decrease in the abundance of *Firmicutes* and a slight decrease in the abundance of *Bacteroidetes*. During and after the GTE treatment, the increased abundance of SCFA-producing bacteria, such as *Ruminococcaceae*.<br><br>The HCM gut microbiota absorbed polyphenols during polyphenol treatment and maintained relatively stable concentrations. Intracellular polyphenol abundance decreased after treatment cessation. 3-methyladenine was significantly increased in the treatment period, which is a PI3-kinase inhibitor and can be stimulated by EGCG.

[147]	Fresh fecal collected from five healthy adults who had not received antibiotics or pre/probiotics in the preceding 3 months	1% (w/v) freeze-dried Laphet, (fermented green tea) ground powder each day	In vitro human fecal fermentation of Lahpet was performed in 300 mL water-jacketed fermenter vessels with a basal growth medium inoculated with 15 mL of fecal slurry (10% PBS). Freeze-dried samples (polyphenol, fiber, polyphenol + fiber) were added at a 1% (w/v) concentration. pH and temperature were maintained at 6.8 and 37 °C under anaerobic conditions with nitrogen gas. Samples were taken at 12 and 24 hours for bacterial enumeration and metabolite analysis. Bacterial diversity was analyzed using 16S rRNA gene sequencing, and metabolite changes were assessed via 1H NMR spectroscopy with Chenomx NMR suite for spectral analysis.	Catechins are relatively stable during human digestion and can reach the colon. Polyphenol compounds and dietary fiber fractions of Laphet differently affect the bacterial abundance at the phylum level.<br><br>In the Polyphenol set, the abundance of *Enterobacteriaceae*, *Proteus*, and *Enterococcus* increased and that of most other genera decreased at 12 or 24 h. In contrast, in the Fiber set, the abundance of *Bacteroidetes*, *Escherichia*-Shigella, *Clostridium sensu stricto* 1, *Phascolarctobacterium*, and *Gammaproteobacteria* increased and that of most other genera decreased at 12 or 24 h. Meanwhile, the abundance of *Bacteroides*, *Enterococcus*, and *GammaProteobacteria* increased and that of *Escherichia-Shigella*, *Fusobacterium*, *ParaClostridium*, and *Clostridiaceae* decreased after 24 h in the polyphenol + fiber set.<br><br>SCFAs produced during in vitro fecal fermentation of *Lahpet* shown difference as well. The concentrations of succinate, and ethanol increased after 24 h fermentation, while those of acetate, butyrate, lactate, and propionate remained unchanged or reduced.

[148]	Gut microbiota culture (*Actinobacteria*, *Bacteroidetes*, *Firmicutes*, *Proteobacteria*, and *Verrucomicrobia*)	0.5 mg/ml GTE dried<br><br>green tea powder (70% ethanol extracts) each day	Each preculture (70 µl) was inoculated into 0.7 ml of fresh BHI broth containing catechin standards (0.2 mg/ml) and GTE (5 mg/ml) in DMSO on a 96-well plate. Gut bacteria were pre-cultured in BHI broth with 0.005% cysteine at 37°C for 48 hours, then divided into three groups based on growth stages: lag, exponential, and stationary. Samples were taken at designated time points, quenched in cold methanol, and stored at -80°C for analysis. Growth curves were constructed to estimate maximum specific growth rates (µmax) for gut microbes with green tea and metabolite extracts. The effects of flavonoids and glucose in GTE were assessed, and flavonoid glycoside concentrations were measured using standard curves. Gut microbial cultures were treated with 0.003 mg/ml GTE.	Four microbial species/strains (*A. equolifaciens*, *E. lenta*, *L. plantarum APsulloc 331261*, and *L. plantarum KACC 11451*) were identified as key players in the biotransformation of GTP by gut microbiota. The biotransformation involved changes in gallic acid, pyrogallol, and C-ring cleavage derivatives.<br><br>Growth modulatory effects of GTP on 37 gut bacteria showed stimulation of *Firmicutes* and *Proteobacteria* growth, inhibition of *Bacteroidetes* and *Actinobacteria,* and varying responses to specific GTF. EGCG inhibited most bacteria, including *L. rhamnosus*, *LimosiLactobacillus reuteri*, *E. coli* and *S. typhimurium*.<br><br>green tea consumption modulated the gut microbiota, and the anti-obesity effects seemed to correlate with changes in the *Firmicutes/Bacteroidete* ratio and alterations in specific bacterial taxa.
[154]	Fecal materials from four healthy volunteers (three males and one female, 24–38 years)	0.1 mmol/L catechin (EC, ECG, EGC, and EGCG)<br><br>(purified) each day	Fecal materials were mixed with a culture medium to simulate human colon conditions. Aliquots of the fecal suspension were spiked with EC, ECG, EGC, and EGCG, then incubated for 48 to 72 hours at 37°C in an anaerobic chamber. UHPLC-MS was used to analyze catechin degradation, and microbiome changes were studied through DNA extraction and high-throughput 16S rRNA gene sequencing.	*Bacteroides* and *Bifidobacterium* were promoted, while potentially harmful bacteria like *Forsterygion varium*, *Bilophila*, and *Enterobacteriaceae* were inhibited by EGCG treatment.<br><br>Microbiota required time to adapt to EGCG presence, seen in changes observed after 12 hours of fermentation.

	[146]	Fecal from ten healthy adult volunteers (six women and four men) aged between 33 and 70 years (average, 47 years)	Participants drank 1000 mL of green tea brew each day	Ten healthy adult volunteers drank about 1000 mL of green tea daily. Fecal samples were collected before, during, and after the 10-day period of exclusive green tea consumption. DNA from these samples was isolated and analyzed using various techniques including PCR amplification, terminal restriction fragment length polymorphism (T-RFLP) analysis, and real-time PCR assays.	The 10-day regimen of drinking green tea caused alterations in the composition of fecal microbiota in most subjects. The results showed an increase in the proportion of bifidobacteria in eight out of ten subjects due to green tea consumption. After cessation of green tea consumption, a decrease in bifidobacteria proportions was observed. There were individual-specific changes in the species composition of bifidobacteria due to green tea consumption.
	[149]	Fecal samples were obtained from 6 healthy volunteers (3 female, 3 males, aged 25-30 years, without recent antibiotic treatment or gastrointestinal disease	1% (w/v) freeze-dried green tea polyphenols (purified) each day	Green tea was extracted and purified to obtain tea polyphenols. Anaerobic fermentation was conducted using fecal samples slurry added to 1350 µL of culture medium (1% GTP) to study the impact of tea polyphenols on gut bacteria and SCFA production. FISH was used to enumerate specific bacterial groups. HPLC was used to quantify short-chain fatty acids produced during fermentation.	Tea polyphenols enhanced *Bifidobacterium* and *Lactobacillus*/*Enterococcus spp*. proliferation but inhibited *Bacteroides–Prevotella* and *Clostridium histolyticum* in gut fermentation.<br><br>Tea polyphenols increased SCFA production, particularly formic and propionic acids, contributing to potential health benefits in the gut ecosystem.
**Oolong Tea**	[150]	Fecal samples were obtained from 6 healthy volunteers (3 female, 3 males, aged 25-30 years, without recent antibiotic treatment or gastrointestinal disease	1% (w/v) freeze-dried oolong tea polyphenols (purified)	Fecal slurries were prepared by mixing fresh fecal samples with autoclaved phosphate-buffered saline (PBS) to create 10% (w/v) suspensions. GTP, OTP, BTP, and FOS (positive control) were combined with autoclaved nutrient basal growth medium to a final concentration of 1% (w/v). Fermentation was initiated by adding 150 µL of fecal slurry to 1350 µL of culture medium, homogenized manually in an anaerobic environment (10% H2, 10% CO2, 80% N2) at 37°C. Samples were collected at 36 hours for metabolite identification. Hybridization was done using 16S rRNA-targeted oligonucleotide probes labeled with Cy3 dye to enumerate specific bacterial groups.	The SCFA concentrations in cultures containing tea polyphenols were significantly increased.<br><br>OTP could significantly increase the *Bifidobacterium* and *Lactobacillus*–Enterococcus spp. and SCFA production, while restrained the proliferation of *Bacteroides–Prevotella* and Clostridim histolyticum groups

	[69]	Fecal samples were obtained from three healthy volunteers (one female and two males, ages 25–30)	100 mg/L oolong tea EGCG、GCG EGCG3"Me (purified) each day	Fermentation was started by adding 150 μL of fecal slurry to 1350 μL of culture medium containing EGCG, GCG, EGCG3"Me, or FOS (positive control) and incubating at 37 °C in an anaerobic chamber. A control group without any added compound was maintained under the same conditions. Samples (250 μL) were collected at 0, 6, 12, and 24 hours for bacterial enumeration by FISH and SCFA analysis using an Agilent 1100 Series HPLC system with a Beckman detector.	Significantly promote the growth of the *Lactobacillus*–Enterococcus group and *Bifidobacterium* spp., while inhibiting the growth of *Bacteroides–Prevotella*, C. histolyticum and Eubacterium–*Clostridium* groups.<br>The total SCFA concentrations were relatively increased compared to that of the control, and the concentrations of formic, acetic, propionic, and butyric acids all increased significantly.
**Black Tea**	[150]	Fecal samples were obtained from 6 healthy volunteers (3 female, 3 male, aged 25-30 years, without recent antibiotic treatment or gastrointestinal disease	1% (w/v) freeze-dried black tea polyphenols (purified) each day	Fecal slurries were made by mixing fresh fecal samples with autoclaved phosphate-buffered saline (PBS) to create 10% (w/v) suspensions. GTP, OTP, BTP, and FOS (positive control) were added to autoclaved nutrient basal growth medium at 1% (w/v). Fermentation was started by adding 150 μL of fecal slurry to 1350 μL of culture medium and incubating in an anaerobic environment (10% H2, 10% CO2, 80% N2) at 37 °C. Samples were collected after 36 hours for metabolite analysis. Hybridization used 16S rRNA-targeted oligonucleotide probes labeled with Cy3 fluorescent dye to identify specific bacterial groups.	The SCFA concentrations in cultures containing tea polyphenols were significantly increased and relatively higher than the control (P < 0.05) at each time<br>BTP could significantly increase the *Bifidobacterium* and *Lactobacillus*–Enterococcus spp. and SCFA production, while restrained the proliferation of *Bacteroides–Prevotella* and *Clostridium* histolyticum groups
	[152]	Two healthy volunteers (one male and one female, aged 22–28 years) who had never had gastrointestinal disease and had not taken antibiotics in the past 3 months.	2% (w/v) black tea polyphenols (70% ethanol extracts) each day	10 g of a 10.0 g/L Keemun and Dianhong black tea solution was mixed with 8 mL of simulated salivary fluid, followed by the addition of salivary α-amylase, CaCl2, and water. After incubating for 2 minutes and adjusting the pH to 3.0, pepsin and gastric lipase were added to simulate gastric juice. The mixture was maintained at 37°C for 2 hours, then the pH was adjusted to 7.0 to simulate intestinal fluid, where pancreatic enzymes and bile salts were added. After 2 hours at 37°C, the mixture was dialyzed and freeze-dried for 48 hours. For in vitro anaerobic fermentation, fresh fecal samples from two healthy volunteers were mixed with physiological saline to create a 10% suspension and used for microbial fermentation. The medium was sterilized at pH 7.0 and fermented in batches with FOS, KBT, and DBT (black tea ingredients), with samples collected for 16S rRNA sequencing analysis.	At the phylum level, Fusobacteria and *Bacteroidetes* were prevalent in the samples collected from the KBT and DBT groups.<br>At the class level, the relative abundances of *Fusobacterial* and *Bacteroidaceae* increased and those of Gamma*proteobacterial*, *Negativicutes*, and Clostridia decreased in the KBT and DBT groups. At the order level, KBT and DBT increased the abundances of *Fusobacteriales* and *Bacteroides* and reduced those of *Clostridiales* , *Selenomonadales*, and *Enterobacteriales*. At the family level, KBT and DBT enhanced the abundances of *Fusobacteriaceae*, *Bacteroidaceae, and* Burkholderiaceae and inhibited those of *Lachnospiraceae*, *Tannerellaceae*, *Acidaminococcaceae*, and *Enterobacteriaceae.*

	[151]	Identical fecal sample from a healthy human volunteer	1000 mg black tea polyphenols each day	The total fermentation time was over 8 weeks, with multiple samplings initiated after steady state I. Beginning after 1 week, SHIME models inoculated with identical fecal samples from a healthy human volunteer, polyphenol extracts, were administered for a 2 week.Finally, a two-week washout period was included at the end of the experiment. A combination of microbiological analyzes including culture, PCR denaturing gradient gel electrophoresis, quantitative PCR, and high-throughput pyrosequencing of the 16S ribosomal RNA gene was applied to characterize microbial community changes.	The response of *Actinobacteria* and *Firmicutes* to single or continuous doses of BTE was significantly reduced. *Bacteroidetes* were not significantly affected by black tea dosage.<br><br>The most significant change at the phylum level was the increase in *Proteobacteria*.<br><br>In addition to individual genera, the relative abundance of the entire family, *Ruminococcaceae*, and *Lachnospiraceae* also decreased with increasing black tea dosage.<br><br>black tea stimulates *Klebsiella*, *Enterococcus* and *Akkermansia* and reduces *Bifidobacterium*, Bacillus coccidioides, *Anaerobic cocci* and Victivallis
	[155]	Fresh human feces were collected from 8 healthy Chinese volunteers (18–24 years old; 4 males and 4 females).	0.1 mg/mL black tea theaflavins (purified) each day	Fecal samples were collected and fermented in vitro with a uniform fecal slurry (HFS) added to General Anaerobic Medium broth. The bacteria were activated through incubation in an anaerobic chamber at 37°C. Sterile TF was added to the suspension, and fermentation was carried out. Samples were collected at 0, 12, 24, and 48 hours, then frozen at -80°C. Genomic DNA from the gut microbiota was extracted, sequenced using 16S rRNA gene amplicons, and processed for data analysis.	At the genus level, a decrease in *Bacteroidetes* and an increase in unidentified *Ruminococcaceae* was observed in TF samples. After 48 h of fermentation, TF significantly increased the relative abundance of unidentified *Ruminococcaceae*, *Clostridium* trichophyton, and Flavonifractor. Eubacterium, unidentified *Ruminococcaceae*, *Clostridium* trichophyton, *Blautia*e species, and flavanols, significantly increased with the fermentation of TF.

Supplementary Table S3: Human feeding trails.

Tea Type	Reference	Subjects	Treatment	Methodology	Major findings
Green Tea	[162]	Fecal from adult volunteers aged between 27 and 46 years. (Healthy, normal weight (BMI 18–24 kg m2), or overweight/obese (BMI >24 kg m2))	400g 7.5g-Tea/L-Water green tea liquid each day	Fecal and saliva samples were collected before and after intervention, with genomic DNA extracted from fecal samples using a standard kit. The V4-V5 regions of the bacterial 16S rRNA gene were amplified by PCR and sequenced on an Illumina HiSeq platform. OTUs and taxonomic affiliations were analyzed, and multivariate analyses (PLS-DA, SIMPER, LEfSe) identified key microbial taxa. DAA assessed taxonomic changes related to GTL consumption, while functional capacity was inferred with PICRUSt and compared using LEfSe.	Green tea consumption significantly altered the overall gut microbiota composition, associated with changes in taxa affiliated with phyla *Firmicutes*, *Bacteroidetes*, *Actinobacteria*, and *Proteobacteria*.<br>Specific microbial families (*Lachinospiraceae*, *Ruminococcaceae*, *Erysipelotrichaceae*, etc.) and genera (*Roseburia*, Faecalibacterium, *Bifidobacterium*, etc.) were notably affected.<br>Notable increases were observed in SCFA-producing bacteria and colonization resistance markers.
	[157]	58 Healthy Caucasians aged 18-50 years, either normal weight (BMI 18–25 kg/m²) or overweight/obese (BMI >25 kg/m²), non-smokers, weight stable, and not on certain medications or specific medical conditions.	GTE capsules (>0.56 g/day EGCG + 0.28 ~ 0.45 g/day caffeine)	The protocol was a randomized, single-blind, placebo-controlled design with two groups: green tea (GT) and placebo (PL). Body composition measurements (weight, BMI, fat mass, waist-to-hip ratio), fecal samples for microbiota analysis, and DNA isolation were conducted at baseline and after 12 weeks.<br>DNA extracted from fecal samples was used for IS-profiling of the gut microbiota using PCR and fragment analysis. Diversity metrics like alpha diversity (within-sample diversity) and beta diversity (between-sample diversity) were calculated and analyzed.<br>clinical trial ID：NCT01556321	Overweight subjects had lower alpha diversity compared to normal weight subjects at baseline. However, the response to GT did not significantly differ between normal weight and overweight subjects in terms of microbial diversity.<br>No significant changes for all phyla combined, *Bacteroidetes*, *Proteobacteria* and dissimilarity in microbial community structure between baseline and week 12 in either GT or PL groups.

[163]	187 healthy postmenopausal women aged 50 - 60 years from the Minneapolis-St. Paul metropolitan area with a body mass index (BMI) between 19.3 and 36 kg/m2 and stable weight were enrolled.	GTE Catechin Complex (Corban complex GTB; Investigational New Drug #103,431) Intake 1315 ± 115.0 mg catechins including 843.0 ± 44.0 mg EGCG (capsules) each day	Participants were randomly assigned to receive either the GTE capsules or identical-looking placebo capsules, twice daily with meals. The daily total intake of the GTE amounted to 1315 ± 115.0 mg total catechins, including 843.0 ± 44.0 mg EGCG. While the placebo capsules contained maltodextrin, cellulose, and magnesium stearate. Fecal samples were collected from participants at the beginning (months 0) and end (months 12) of the study and were prepared by sonicating and centrifuged to obtain supernatants for further analysis.	The relative abundances of *Firmicutes*, *Bacteroidetes*, and *Actinobacteria*, the dominating phyla in fecal microbiome did not differ between groups.<br>No significant differences were observed on class, family, and genus levels.
[104]	A total of 85 participants (65.9% male; mean age: 43.3 years) without T2DM were included.	The mean (SD) intakes of total green tea were 443 (417) mL/day, respectively. The median intakes of catechins and EGCG were 67.8 (23.2–150.1) and 9.5 (0.9–26.9) mg/day (green tea brew)	85 individuals without T2DM were recruited over four seasons. Blood samples, stool samples, 3-day weighed dietary records, and green tea samples were collected across seasons. Dietary intake, lifestyle factors, anthropometric measurements, and biochemical markers were assessed. Stool samples underwent DNA extraction and 16S rRNA gene sequencing for microbial analysis. Regression models were used to assess associations between green tea intake, gut microbiota, and glucose metabolism biomarkers. Mediation analysis was conducted to assess the role of the gut microbiota as a mediator.	Green tea intake was linked to alterations in the relative abundance of specific gut microbial species. Positive associations were observed with certain species like *B. ovatus* and *Flavonifractor plautii*, while negative associations were seen with others like *Phocaeicola vulgatus*.<br>*P. vulgatus* partially mediated the association between green tea intake and fasting blood glucose levels.
[164]	20 individuals with MetS and 20 age- and gender-matched healthy persons.	GTE 1g containing 890 mg of total catechins) confections each day	20 individuals with MetS and 20 matched healthy controls will receive 1 g/day of green tea extract (GTE) containing 890 mg catechins or a placebo for 4 weeks, followed by a 1-month washout. Blood samples will be collected on days 0, 14, and 28 for metabolic assessments, RNA isolation, and safety monitoring. Fecal samples and a gut permeability test will be conducted on day 28. The study will also include dietary records, anthropometrics, and blood pressure measurements at various times. The primary outcome is serum endotoxin levels, while secondary outcomes include gut microbiota composition, fecal SCFAs, intestinal permeability, inflammatory markers, and expression of TLR4/NFκB signaling genes.<br><br>clinical trial ID：NCT03973996	GTE is expected to decrease serum endotoxin in a time-dependent manner and to a greater extent in MetS persons due to their underlying metabolic endotoxemia.<br><br>GTE decreases gut-derived endotoxin translocation by improving gut barrier integrity and/or decreasing pathogenic LPS-containing Gram-negative bacteria populations (e.g. *Proteobacteria*) of the gut microbiota

Black Tea	[161]	Fecal from 72 Healthy males or females between 20 and 59 years of age	3 cup of Black tea brew (polymerized polyphenols 76.2 mg) each day	Tea bags with 2.0 kg of tea leaves were used for 150 L of boiling water, with 90 seconds of steeping. Participants consumed the tea three times, totaling 12 times. Gut microbiota analysis was performed using a sequencing-based method to determine absolute and relative values of bacterial groups by measuring logarithmic copies of each species.<br><br>clinical trial ID: UMIN000038168	Black tea consumption for 12 weeks had a significant effect on the abundance of specific bacteria in the gut. In subjects with low amounts of *Flavonifractor plautii*, black tea intake increased the absolute amount of this bacterium. *Flavonifractor plautii* has been reported to have beneficial effects on immune function.<br><br>Black tea intake also increased the abundance of *Prevotella*, a group of bacteria known for their ability to produce acetic acid. Additionally, black tea intake increased the abundance of butyrate-producing bacteria in subjects with low salivary mmunoglobulin A （SIgA） concentration.
	[160]	Fecal from human volunteer	1 cup of black tea brew (made from dry tea powder) each day (There is no prescribed time or dosage for drinking black tea)	Volunteers participated in a randomized, double-blind, crossover study comparing black tea with a placebo. After a two-week break-in period, there was a 4-week washout during which alcohol and tea were prohibited. During both treatment periods, volunteers followed the same control diet. Stool samples were collected on days 1, 14, and 21 of each intervention, totaling 6 samples per subject. Samples were kept on ice and processed for FISH analysis to study microbial communities.	None of the black tea interventions resulted in a statistically significant difference in the number of bacteria hybridizing to a specific probe.<br><br>During black tea consumption, reduced numbers of "other bacteria" were detected, which were detected with the general eubacteria probe but not with any of the more specific probes. Drinking black tea did inhibit the growth of certain bacteria in the fecal microbiota.
Oolong Tea	[159]	Fecal were obtained from 28 healthy adults ranging in age from 20 to 50 years, whose weight had been stable for at least 3 years.	500ml 5 mg/ml oolong tea water brew (Total phenolic content 636.17±8.54 µg GAE/mL) each day	Subjects' body composition was assessed before and 3 weeks after treatment, with fecal samples collected for gut microbial and metabolic analysis. The V3 and V4 regions of the 16S rRNA gene were sequenced, and a genomic paired-end library was constructed and sequenced. The data were processed through filtering, trimming, merging, denoising, and clustering, followed by taxonomic assignment, species abundance analysis, and evaluation of community diversity and group differences.<br><br>clinical trial ID: NKUIRB2022085	3-week continuous oolong tea supplementation increased the abundance and diversity of gut microbial communities from the examined subjects. At the phylum level, *Firmicutes* and *Bacteroidetes* were predominant in all samples. Although oolong tea intervention inhibited *Firmicutes* and *Proteus* and slightly promoted *Bacteroides*, the differences between groups are not statistically significant. the relative abundance of *Megamonas* was remarkably inhibited by oolong tea intervention compared with baseline at the genus level, whereas *Bacteroides* and *Prevotella* were detected at remarkably higher abundances in the gut microbial profile from subjects treated by 3-week oolong tea intervention with statistical differences.

Pu-erh tea	[159]	Thirteen healthy male volunteers, age ranged from 24 to 32 year	300 mL Pu-erh tea brew (powder) (50 mg/Kg) each day	Participants consumed 300 mL of instant Pu-erh tea infusion twice daily for 4 weeks at a dose of 50 mg/kg/day. Standard meals were provided for one week before the tea intervention. Serum and fecal samples were collected at the end of the pre-intervention week (Pre-Tea) and after the fourth week of tea consumption (Post-Tea), before the first meal. DNA was extracted from bacterial samples, followed by PCR amplification of the 16S rRNA gene V4–V5 region with barcodes for sequencing. PCR amplicons were purified, quantified, pooled, and sequenced. Sequencing data were processed for quality filtering, chimera detection, OTU clustering, taxonomic classification, and OTU table generation.	Pu-erh tea induced changes in the structure of human fecal microbial communities, with a decrease in the relative abundance of *Bacillus* and *Clostridium* in human fecal.<br><br>Pu-erh tea-induced microbial changes in mice and human showed the same tendencies at the Phylum and Class levels. Some of the relative abundance changes at the genus level were also identified. In human stool samples, the microbiota changes at the genus level showed that the relative abundances of OTUs in *Lactobacillus, Bacillus, Streptococcus* and *Lactococcus genera* were reduced by tea intervention.<br><br>The activity of bile-salt hydrolase was significantly reduced in the fecal samples of human subjects in the post-tea group.
